# A multi-centered trial investigating gestational treatment with ursodeoxycholic acid compared to metformin to reduce effects of diabetes mellitus (GUARD): a randomized controlled trial protocol

**DOI:** 10.1186/s13063-022-06462-y

**Published:** 2022-07-19

**Authors:** Holly Lovell, Alice Mitchell, Caroline Ovadia, Noelia Pitrelli, Annette Briley, Claire Singh, Hanns-Ulrich Marschall, Kennedy Cruickshank, Helen Murphy, Paul Seed, Catherine Williamson

**Affiliations:** 1 Guy’s and St Thomas’ NHS Foundation Trust, London, UK; 2 King’s College London, London, UK; 3 Caring Futures Institute, Flinders University South Australia, Adelaide, Australia; 4 University of Gothenburg, Gothenburg, Sweden; 5 University of East Angela/ Cambridge University Hospitals NHS Foundation Trust, Cambridge, UK

**Keywords:** Gestational diabetes mellitus, Treatment, Ursodeoxycholic acid, Metformin, Randomized clinical trial

## Abstract

**Background:**

Each year in the UK, approximately 35,000 women develop gestational diabetes mellitus (GDM). The condition increases the risk of obstetric and neonatal complications for mother and child, including preeclampsia, preterm birth, and large for gestational age babies. Biochemical consequences include maternal hyperglycemia, neonatal hypoglycemia, and dyslipidemia. Metformin is the most commonly used firstline pharmacological treatment. However, there are concerns about its widespread use during pregnancy, due to its limited efficacy and potential safety concerns. Therefore, there is a need for additional therapies that improve both maternal–fetal glucose and lipid metabolism.

Ursodeoxycholic acid (UDCA) is not currently used for treatment for GDM. However, it can improve glucose control in type 2 diabetes, and it improves fetal lipid profiles in gestational cholestasis. Consequentially, it is hypothesized that treatment with UDCA for women with GDM may improve both maternal metabolism and neonatal outcomes. The primary outcome of this trial is to assess the efficacy of UDCA compared with metformin to improve glucose levels in women with GDM.

**Methods:**

The trial is a two-armed, open-label, multi-center, randomized controlled trial. Women are eligible if they have been diagnosed with GDM by an oral glucose tolerance test between 24 + 0 and 30 + 6 weeks’ gestation, and if they require pharmacological intervention. In total, 158 pregnant women will be recruited across seven NHS Trusts in England and Wales. Women who consent will be recruited and randomized to either metformin or UDCA, which will be taken daily until the birth of their baby. Maternal and neonatal blood samples will be taken to evaluate the impact of the treatments on maternal glucose control, and maternal and neonatal lipid metabolism. Maternal and fetal outcomes will be evaluated, and acceptability of UDCA compared with metformin will be assessed.

**Discussion:**

This trial has the potential to identify a potential new treatment for women with GDM. If successful, a future large multi-center trial will be designed to investigate where decisions can be personalized to identify which women will respond more effectively to UDCA than alternatives to improve maternal and baby outcomes.

**Trial registration:**

ClinicalTrials.gov NCT04407650.

**Supplementary Information:**

The online version contains supplementary material available at 10.1186/s13063-022-06462-y.

## Administrative information

Note: the numbers in curly brackets in this protocol refer to SPIRIT checklist item numbers. The order of the items has been modified to group similar items (see http://www.equator-network.org/reporting-guidelines/spirit-2013-statement-defining-standard-protocol-items-for-clinical-trials/).

**Table:** 

Title {1}	A multi-centered trial investigating **G**estational treatment with **U**rsodeoxycholic **A**cid compared to Metformin to **R**educe effects of **D**iabetes mellitus (GUARD): a randomized controlled trial protocol Acronym: GUARD
Trial registration {2a and 2b}.	Clinicaltrials.gov – NCT04407650
Protocol version {3}	21/10/2021 Version 4.0
Funding {4}	Funded by J.P. Moulton Foundation and Professor Catherine Williamson’s NIHR Senior Investigator grant
Author details {5a}	Catherine Williamson, Caroline Ovadia, Paul Seed, Alice Mitchell, Kennedy Cruickshank, Claire Singh – King’s College London Helen Murphy – University of East Angela/ Cambridge University Hospitals NHS Foundation Trust Annette Briley – Flinders University South Australia Hanns-Ulrich Marschall – University of Gothenburg Holly Lovell, Noelia Pitrelli,– Guy’s and St Thomas’ NHS Foundation Trust
Name and contact information for the trial sponsor {5b}	Co-Sponsors: King’s College London – Amy Holton amy.holton@kcl.ac.uk Guy’s and St Thomas’ NHS Foundation Trust – Rachel Fay r&d@gstt.nhs.uk
Role of sponsor {5c}	The funder had no role in the design of the study, and will not be involved in the collection, management, analysis, or interpretation of the data, nor the final report. The sponsor is responsible for ensuring the study is conducted in line with Good Clinical Practice (GCP), and that procedures and arrangements are in place and adhered to for monitoring the research to ensure safety. The sponsor will not be involved in analysis or interpretation of data and will not have authority over the final report.

## Introduction

### Background and rationale {6a}

Each year in the UK, approximately 35,000 women develop diabetes during pregnancy, a condition called gestational diabetes mellitus (GDM). This increases the risk adverse pregnancy complications and has future health implications for both mother and child [1]. Antenatal maternal complications include an increased risk of hypertensive diseases of pregnancy, including preeclampsia [2], and higher rates of cardiovascular disease and type 2 diabetes mellitus (T2DM) in later life [3–5]. Aside from hyperglycemia, GDM is further complicated by maternal dyslipidemia; triglyceride and free fatty acid concentrations are increased in maternal blood, while high-density lipoprotein (HDL)-cholesterol is reduced [6, 7]. Metabolomic studies show disturbances in intermediary lipid metabolites, e.g., acyl-carnitines and phospholipids [7, 8]. Early decline in plasma adiponectin, an indicator of poorer mitochondrial oxidation in overweight and obese women, is an almost universal finding in GDM pregnancy regardless of maternal BMI [9]. Thus, GDM is a potentially vasculotoxic condition, associated with abnormal lipid and glucose metabolism [10].

GDM is associated with accelerated fetal growth and increased risk of the baby being large for gestational age (LGA), defined as birth weight above the 90th percentile for sex and gestational age [1, 10]. It is also complicated by higher rates of preterm birth, caesarean section, and birth injuries, including shoulder dystocia, which is particularly increased with LGA [1, 2, 11]. Due to the complications of preterm birth and hyperinsulinemia, GDM offspring are more likely to require admission to neonatal intensive care units for treatment of hypoglycemia, jaundice, and respiratory distress [12]. GDM causes fetal dyslipidemia, with increased free fatty acids and triglycerides in the umbilical cord blood; this is also associated with increased risk of LGA [13–15]. The children of women with GDM have increased rates of obesity, childhood cardiovascular disease, and T2DM in later life, likely related to exposure both to hyperglycemia and hyperlipidemia in utero [16, 17].

#### Effectiveness of current treatments

In the UK, women with risk factors for GDM typically have a 75-g oral glucose tolerance test (OGTT) at 24–28 weeks’ gestation. Those that test positive (fasting glucose concentration ≥ 5.6 and/or 2-h ≥ 7.8 mmol/L) start self-monitoring of blood glucose (SMBG) and are given dietary and lifestyle advice [18]. If unable to achieve the National Institute for Health and Care Excellence (NICE)-recommended glucose control targets (fasting glucose < 5.3, 1 h < 7.8 mmol/L and/or 2 h < 6.7 mmol/L), they are offered either a pharmacological oral glucose-lowering medication, metformin, or subcutaneous insulin injections. Metformin is the most commonly used firstline pharmacological treatment. However, concerns have been raised about its widespread use during pregnancy, both because of its limited efficacy, and due to potential safety concerns. Metformin crosses the placenta, has growth inhibitory properties, and suppresses mitochondrial respiration, all of which could, theoretically, adversely affect the developing fetus [19, 20]. The Metformin in Gestational Diabetes (MiG) trial demonstrated that mothers randomized to metformin, compared with insulin, had reduced maternal weight gain and were less likely to develop gestational hypertension [21]. However, the rate of LGA was not affected, and the offspring had more subcutaneous fat at 2 years of age than those randomized to insulin [22]. A study of maternal metformin treatment for pregnant women with polycystic ovary syndrome also did not show an impact on LGA [23], and the offspring were heavier at both 1 and 4 years of age, compared to those whose mothers were randomized to a placebo [24]. However, a separate study of 4-year-old children of pregnant women with obesity, who had been randomized to either metformin or placebo, demonstrated improved cardiovascular parameters and no adverse impact on body composition [25].

Thus, some current data have raised concerns that metformin, currently used by many women with GDM, does not adequately prevent outcomes such as LGA and may have negative long-term effects on the metabolic health of the children [26, 27]. The impact of metformin on childhood body composition in some studies may be, at least in part, because metformin has less effect on serum triglyceride concentrations than insulin [21]. It is noteworthy that in the MiG trial, metformin alone was inadequate for achieving glycemic targets in approximately 50% of women, necessitating supplementary treatment with insulin [21]. Indeed, even insulin treatment (the “gold standard” pharmacological approach) was not shown to be of definitive benefit for GDM offspring in a recent Cochrane review [28].

Glibenclamide is another other oral hypoglycemic agent that has been used to treat GDM. However, it has not been shown to be superior to insulin treatment in randomized trials [29], or as an add-on therapy to metformin [30]. Therefore, there is an urgent unmet need for additional therapies that improve maternal–fetal glucose and lipid metabolism, and the longer-term health outcomes of women who develop GDM and GDM-exposed offspring.

#### Rationale for the use of ursodeoxycholic acid (UDCA)

UDCA is currently not an established/licensed treatment for GDM. However, a meta-analysis, including data from 7 trials, showed that UDCA improves fasting glucose, insulin, and HbA1c concentrations in non-pregnant individuals [31]. Furthermore, pilot data from studies of UDCA treatment of women with intrahepatic cholestasis of pregnancy (ICP) have demonstrated reduced insulin resistance, indicating UDCA has the potential to be an effective treatment to improve maternal glycemia in GDM [32]. UDCA is commonly used in pregnancy for the treatment of ICP, and a recent randomized, placebo-controlled trial investigating ICP treatment and management did not show any increase in adverse events, including gastrointestinal symptoms, in women treated with UDCA compared with placebo [33].

Women with ICP have increased rates of GDM (odds ratio 2.81, 95% CI 2.32–3.41) [34]. Continuous glucose monitoring demonstrated ICP-associated elevations in prandial glucose concentrations, abnormal glucose tolerance, and reduced secretion of the gut hormone glucagon-like peptide-1 (GLP-1) [35], which acts to enhance glucose-mediated insulin secretion [36]. Improvement in GLP-1 in women with ICP treated with UDCA has been shown [35]. Of note, GLP-1 is thought responsible for 70% of the insulin release following meals, and levels are lower in women with GDM than unaffected pregnancies [37].

Women with ICP have dyslipidemia in addition to increased susceptibility to ICP-associated GDM. This is characterized by elevated serum concentrations of total cholesterol and low-density lipoprotein (LDL) cholesterol. UDCA treatment has been shown to improve fetal serum lipid parameters [32]. Consequentially, UDCA may be more effective than metformin at reducing the frequency of LGA infants in women with GDM.

There is increasing evidence that the gut microbiota plays a role in maternal glucose and lipid metabolism [38–40]. Studies of the gut microbiota in T2DM have reported reductions in butyrate-producing bacteria in untreated compared to metformin-treated patients [41]. Butyrate is a metabolically active short-chain fatty acid (SCFA), increased levels of which are associated with improved glycemia [42]. UDCA is metabolized by gut bacteria, and likely also influences composition of the gut microbiota [43]. We hypothesize that this will result in increased release of gut hormones (GLP-1 and FGF19) that improve maternal/fetal blood concentrations of lipids, e.g., triglycerides, glucose, and reduce rates of obstetric and neonatal complications. Pilot data support this hypothesis from studies of women with intrahepatic cholestasis of pregnancy (ICP) treated with UDCA [32].

Treatment with UDCA over 12 weeks was shown to reduce weight and HbA1c (glycosylated hemoglobin) in a small study in patients with T2DM and hepatic impairment [37]. Furthermore, a recently published meta-analysis of studies in people with nonalcoholic fatty liver disease (a disorder that is commonly associated with T2DM and with risk increased in those with previous GDM) [44] reported that UDCA treatment was associated with significant reduction in fasting glucose, HbA1c, and plasma insulin concentration. UDCA is therefore a biologically plausible treatment, but it has not yet been evaluated in GDM [31]. Table 1 summarizes the mechanisms by which UDCA and metformin influence glucose and lipid metabolism.

**Table 1:** Proposed mechanisms of action of UDCA and metformin in GDM

	UDCA	Metformin
Inhibition of the mitochondrial respiratory chain complex 1; leads to activation of hepatic AMPK, reducing SRBEP1c which controls glucose-stimulated genes associated with lipid, glucose, and protein formation, and stimulates fatty acid oxidation and glucose uptake	X	✓
Activation of hepatic FXR, reducing SRBEP1c which controls glucose-stimulated genes associated with lipid, glucose, and protein formation, and stimulates fatty acid oxidation and glucose uptake	✓	X
Brown adipose tissue activation of AMPK, breakdown of VLDL-TG, mitochondrial content	X	✓
Brown adipose tissue signaling via TGR5 to increase energy expenditure by increasing UCP1	✓	X
Increased skeletal muscle insulin sensitivity and insulin-mediated glucose uptake	X	✓
GLP-1 receptor increase and reduced GLP-1 breakdown	X	✓
GLP-1 release increase	✓	X
Reduction of endoplasmic reticulum stress in obese individuals, reducing insulin resistance	✓	X

It is important and timely to evaluate the impact of UDCA on maternal and fetal outcomes in GDM. The GUARD trial will therefore compare the impact of treatment with UDCA to metformin on glycemic control in women with GDM.

### Objectives {7}

#### Primary objective

The primary objective is to assess the efficacy of UDCA, compared to metformin, to improve glycemic control in women with GDM.

#### Secondary objectives

The secondary objectives are to:

- Evaluate the impact of each treatment on maternal and neonatal lipid metabolism.
- Assess the acceptability of UDCA compared to metformin for women with GDM.
- Establish whether continuous glucose monitoring gives clinically useful information in the overall assessment of maternal glycemic control in women with GDM.
- Compare maternal and fetal outcomes of relevance to treatment with UDCA or metformin.
- Evaluate the vascular response in each arm—an optional element of the trial for participants.

### Trial design {8}

GUARD is a phase IV two-armed, open-label, multi-centered, randomized controlled trial. Participants are randomized with a 1:1 allocation ratio. Randomization will be minimized in groups by four variables: BMI, previous history of GDM, severity, and by center.

## Methods: participants, interventions and outcomes

### Study setting {9}

Participants will be recruited from antenatal clinics in maternity units across seven NHS Trusts in England and Wales.

### Eligibility criteria {10}

The inclusion criteria are:

1. Women between 16 and 50 years of age with GDM diagnosed between 24^+0^ and 30^+6^ weeks’ gestation in accordance with the NICE guidelines (one or more glucose concentrations of ≥ 5.6 mmol/l fasting or ≥ 7.8 mmol/l 2 h after a standard 75 g OGTT) and requiring pharmacological treatment.
2. Booking BMI ≥ 18.5 kg/m^2^.
3. Planned antenatal, intrapartum, and postpartum care at the participating center.

The exclusion criteria are:

1. Unwilling/unable to give written informed consent and comply with the requirements of the study protocol.
2. Multifetal pregnancy.
3. Congenital anomaly on ultrasound requiring fetal medicine input.
4. Previous diagnosis of diabetes outside of pregnancy.
5. HbA1c at booking ≥ 48 mmol/mol or ≥ 6.5% during current pregnancy (if available).
6. Significant pre-pregnancy comorbidities that increase risk in pregnancy, for example renal failure, severe liver disease, transplantation, cardiac failure, psychiatric conditions requiring in-patient admission (within previous year), in the opinion of the responsible clinician or the Chief Investigator (CI).
7. Significant co-morbidity in the current pregnancy, i.e., physical or psychological conditions likely to interfere with the conduct of the study and/or interpretation of the trial results in the opinion of the responsible clinician or the CI.
8. Not fluent in English and absence of interpreter or translation services for all study visits.
9. Participation in another intervention study where the results could influence GDM-related endpoints, in the opinion of the responsible clinician or the CI, or participation in a CTIMP during current pregnancy.
10. Known allergy/hypersensitivity/intolerance to the active substance or excipients, or patients taking any medications that are contraindicated as per the Investigational Medicinal Product (IMP) Summary of Product Characteristics (SmPC).

### Who will take informed consent? {26a}

Women who have been diagnosed with GDM by a routine OGTT will be identified by the clinical team and given the patient information sheet (PIS), alongside an opportunity to discuss the trial with the research team. The clinical team will identify women who need to commence pharmacological treatment between 24 weeks and 32 + 6 weeks gestation and will then offer the trial and refer to the research team women who wish to participate.

Written informed consent will be gained by trained and delegated members of staff (research or clinical). For women who are being seen face to face, the informed consent form (ICF) will be signed and dated by both parties during the visit. Due to changes in the clinical pathway in response to the COVID pandemic, many diabetes clinic appointments are now virtual; consequently, there is also the option for potential participants to provide informed consent remotely. During the remote consent process, the delegated investigator will confirm the participant understands and is happy to consent to each point on the consent form, and then will sign, date, and time stamp part 2 of the consent form. The participant will have a copy of the ICF at home and will concurrently sign, date, and time stamp part 1. A research midwife will then perform a home visit to deliver the IMP and perform baseline assessments. Consent will be confirmed during this visit, with the participant countersigning the clinician signed ICF. The participant will be given a copy of the ICF signed by all parties in addition to the PIS.

### Additional consent provisions for collection and use of participant data and biological specimens {26b}

Participants have the option to consent to their samples being stored for future appropriately reviewed and approved research. This is indicated on the ICF by the participant initialing “Yes” or “No” for this question.

## Interventions

### Explanation for the choice of comparators {6b}

NICE guidelines recommended metformin as the firstline medication for women who are unable to achieve their blood glucose targets through lifestyle interventions alone, and although not licensed for use in pregnancy, it is standard care across the UK [18]. Metformin is recommended prior to insulin due to greater acceptability, ease of administration, yet the clinical effectiveness is comparable; it is therefore most suitable as a comparator.

### Intervention description {11a}

Participants will be randomized in equal proportions to either UDCA or metformin. Each drug will be supplied, re-packaged, labelled, and distributed by the Pharmacy Manufacturing Unit at Guy’s and St Thomas’ NHS Foundation Trust (GSTFT PMU). In both arms, participants will take the first dose within 2 days of the baseline visit and will continue self-administration at home, while they undergo regular glucose checks, in line with current clinical practice. The randomized treatment medication will be taken daily until delivery.

The glucose control targets follow NICE pregnancy guidelines, aiming to maintain all capillary glucose levels between 3.9 and 7.8 mmol/l. The specific pre- and post-prandial SMBG targets are ≤ 5.3 mmol/L before breakfast, ≤ 7.8 mmol/L 1-h post meal, and ≤ 6.7 mmol/L 2-h post meal. All participants will receive antenatal education regarding diet and lifestyle as part of their standard clinical care pathway. In each arm, insulin may be added as a rescue medication if oral treatment does not control blood glucose levels, in accordance with standard antenatal clinical practice.

#### UDCA

UDCA 500-mg film-coated tablets (Ursofalk®, Dr Falk) will be packed into packs of 28 tablets (2 weeks’ supply). Participants randomized to UDCA will take 500 mg twice a day orally with the morning and evening meal.

#### Metformin

Metformin 500-mg tablets (Medley) will be packed into packs of 56 tablets (2 weeks’ supply). Participants randomized to metformin will be started following a dose escalation scheme to minimize side effects, until a dose of 1000 mg BD is reached:

- Days 1 and 2: 500 mg with evening meal
- Days 3 and 4: 500 mg with breakfast and 500 mg with evening meal
- Days 5 and 6: 500 mg with breakfast and 1000 mg with evening meal
- Day 7 and remaining: 1000 mg with breakfast and 1000 mg with evening meal

### Criteria for discontinuing or modifying allocated interventions {11b}

Participants have the right to withdraw from the trial at any time, without giving a reason. All data (information/ samples) collected up until the woman withdrew will be used, unless she specifically requests otherwise. In which case, all information and samples will be withdrawn from the study dataset. There is also an option to withdraw from the study drug only. In this scenario, the participant will be asked to confirm whether they are still willing to provide prospective trial-specific data, research samples, or data collected as per routine clinical practice.

The investigator may decide to discontinue the intervention in the case of concurrent illnesses or any adverse events; however, these will be assessed on a case-by-case basis and following discussion with the Chief Investigator and central research team.

### Strategies to improve adherence to interventions {11c}

Participants are asked to complete a diary card, documenting each time they take a dose of their IMP. The cards will be reviewed to assess adherence at each study visit and missed doses will be recorded on the trial database’s electronic case report form (eCRF). If a participant forgets to return this card, or has not completed it, they will be asked if they have missed any doses, and this will be recorded as a percentage. The research midwife will contact participants prior to follow-up visits as a reminder to attend and bring the card with them.

### Relevant concomitant care permitted or prohibited during the trial {11d}

A complete list of concomitant medication taken between randomization and birth, excluding medications during labor, will be recorded on the eCRF. Insulin may be added as a rescue medication if oral treatment alone does not control a participant’s blood glucose levels. This will be prescribed and administered as per standard antenatal practice and will be considered a Non-Investigational Medicinal Product (nIMP).

### Provisions for post-trial care {30}

It is not anticipated that there will be any requirements for post-trial care.

### Outcomes {12}

#### Primary outcome

##### To assess the efficacy of UDCA compared with metformin to improve glycemic control in GDM

Efficacy will be measured by comparing maternal fasting blood glucose concentration at 36 weeks’ gestation, ± 1 week, between the two groups. Multiple regression will be used to adjust for baseline fasting glucose, BMI, and whether the participant has previously had GDM.

This gestation was chosen due to pragmatic benefits; it coincides with scheduled clinical appointments and routine delivery would not be planned prior to this stage of pregnancy.

A meta-analysis examining the effect of UDCA on glycemic markers also used fasting blood glucose as a marker to demonstrate the efficacy of UDCA on glycemic control [31].

#### Secondary outcomes

##### Evaluation of the impact of each treatment on maternal and neonatal lipid metabolism

Maternal lipid metabolism is assessed at 36 weeks by fasting measurement of blood triglyceride, total cholesterol, calculated LDL cholesterol, HDL cholesterol, and free fatty acid concentrations.

Neonatal lipid metabolism is assessed through analysis of umbilical cord blood, measuring C-peptide, triglyceride, total cholesterol, calculated LDL cholesterol, HDL cholesterol, and free fatty acid concentrations.

We hypothesize that if improved lipid control is demonstrated, this may result in a reduction of LGA babies born to women treated with UDCA. Pilot data in women with ICP demonstrates that treatment with UDCA reduced the concentration of free fatty acids and other lipids in umbilical venous blood [32].

##### Acceptability of UDCA compared to metformin to women with GDM

Acceptability of treatment will be assessed using two validated questionnaires: Quality of Life questionnaire (EQ-5D-5L) [45] and the Diabetes Treatment Satisfaction questionnaire (DTSQ) [46]. Participants will be asked to complete the EQ-5D-5L at baseline and at 36 weeks, and the DTSQ will be completed at 36 weeks. Mean scores for each group will be reported.

##### To establish whether continuous glucose monitoring gives clinically useful information for the assessment of maternal glycemic control in women with GDM

Each participant will have a continuous glucose monitor (CGM) device inserted at baseline, follow-up 1, and follow-up 2. The device will be worn for 10 days each time. The CGM measures interstitial glucose concentration every 5 min through a sensor that is placed subcutaneously. With 288 glucose measurements/day, CGMs provide detailed glucose information about overnight and post-prandial glucose concentration, providing direct insight into fetal exposure to maternal glycemia [47]. A recent large international consensus paper highlighted CGM as a robust research tool and emphasized the accuracy of contemporary sensors, the detailed information they provide, and non-invasive nature compared to frequent capillary glucose monitoring [48].

The data collected from the CGM will determine the percentage of time spent within target (glucose concentrations 3.9–7.8 mmol/L), the percentage time spent above and below target measures of glucose variability including glucose standard variation (SD), co-efficient of variation (CV), and frequency and duration of glycemic excursions measured by the area under the curve (AUC) for the pre-specified glucose thresholds.

##### To compare maternal and fetal outcomes that could relate to treatment with UDCA or metformin

The following outcomes will be obtained from participants’ clinical records and include:

- Mode of birth (rates of caesarean section (CS), elective and emergency procedures, assisted vaginal birth and spontaneous vaginal delivery (SVD).
- Gestational age at birth.
- Apgar score @ 5 min post birth.
- Occurrence of shoulder dystocia,
- Infant birth weight (customized birth weight percentile, calculated using the GROW centile calculator [49], proportion of babies born large for gestational age (LGA), and proportion of babies born small for gestational age (SGA).
- Neonatal morbidity (treatment for neonatal hypoglycemia, neonatal jaundice, respiratory distress, or birth trauma).
- Neonatal intensive care and special care unit admission (number of inpatient nights).
- Perinatal death: fetal demise, stillbirth, or neonatal death.
- Biochemical analysis of maternal blood for liver function tests at follow-up 2 (ALT, bilirubin, ALP), bile acid, and C-reactive protein concentrations.
- HbA1c concentration; a conventional marker of medium-term glycemia (except at follow-up 1).
- Proportion of women requiring rescue medication, insulin treatment (time until treatment addition and maximum daily total dose of insulin required).
- Maternal gestational weight at 36 weeks compared with weight at first trimester screening visit.
- Estimated blood loss at time of birth.

Outcomes assessed through samples analyzed by the research team include:

- Serum concentration of 1,5-anhydroglucitol; a novel marker of short-term glycemia [1, 4]
- Optional maternal stool sample to investigate the effect of UDCA/metformin on maternal gut microbes and metabolites that can influence maternal metabolism.
- Fetal exposure to UDCA/metformin assessed by analysis of a neonatal meconium sample.

##### To evaluate vascular responses in each arm (optional element)

A calibrated cuff-based blood pressure instrument, the arteriograph, will be used to obtain vascular readings, as recently used in a maternal hypertension trial [50]. Vascular health is included as a secondary outcome, as arterial function measures are more powerful than, and independent of, standard blood pressure for later prognosis [51–53]. The arteriograph works by a minor supra-systolic inflation so that the cuff senses the waveform from each heartbeat for 4–6 beats, thereby providing both a blood pressure measure and a measure of arterial stiffening by sensing the waveform recorded. It has a British Hypertension Society A/A grading for the accuracy of its blood pressure measurement. Arteriograph measurements will be performed only in participants who provide additional consent to this optional element. In those who agree, it will be undertaken at baseline, follow-up 1, and follow-up 2. The measurements recorded include (i) maternal pulse wave velocity, with systolic and diastolic blood pressure, (ii) central arterial pressure, and (iii) augmentation index.

### Participant timeline {13}

Table 2 summarizes the participant’s pathway through the trial. It is expected that each participant will be in the treatment period for a maximum of approximately 16 weeks, with the result of the HbA1c sample collected 3 months post birth from the local GP. The end of the trial will be defined as when the database is locked, all recruits have completed all the study-related visits, and the data has been entered in the eCRF and cleaned.

**Table 2:** Participant timeline

Visit name and approximate pregnancy week	Participant identification	Baseline	Follow-up 1	Follow-up 2 *	Birth	Post birth
	24^+0^–30^+6^	24^+0^–32^+6 a^	32^+0^ ± 1 ^a^	36^+0^ ± 1 ^b^		3 month post birth^c^
Patient information	X ^h^					
Informed consent		X				
Inclusion / exclusion criteria		X				
Demographics		X				
Medical and obstetric personal and family history		X				
Adverse events		X	X	X	X ^m^	
Concomitant medication		X	X	X	X ^n^	
Weight		X^t^	X^t u^	X		
Height		X				
Blood pressure and pulse ^d^		X	X^u^	X		
Fasted glucose	X ^s^			X		
HbA1c	X ^i^	X ^j^		X		X ^r^
Liver function tests, bile acids and C-reactive protein, U&E		X		X		
Lipid profile				X		
1,5-Anhydroglucitol and non-fasting metabolic hormones^e^		X	X^u^			
1,5-Anhydroglucitol and fasting free fatty acids and metabolic hormones ^e^				X		
Cord blood samples ^e^					X	
Blood spots ^e^					X ^o^	
Meconium collection ^e^					X	
Randomization		X				
IMP dispensing		X	X	X		
IMP administration		X (continuously)				
Dispense diary card		X				
Drug diary review			X	X	X	
Continuous Glucose Monitoring ^f^		X	X^u^	X		
Download CGM data			X^u^	X	X	
Quality of life questionnaires (EQ-5D-5L)		X		X		
Treatment satisfaction questionnaires (DTSQs)				X		
4-day food diary		l	l	X ^l^		
Labor and birth data					X ^p^	
Neonatal birth weight					X ^p^	
Neonatal data					X ^p q^	
**Optional** vascular studies ^g^		X	X^u^	X		
**Optional** fecal sample ^e^				X ^k^		

^a^Follow-up visits will be adjusted to ensure the participant has been receiving IMP for at least 2 weeks. Follow-up 1 will not be required when the baseline visit occurs after 31 + 6 weeks gestation

^b^Women must fast for at least 3 h

^c^To occur at local GP practice

^d^Blood pressure in triplicate and pulse for women who do not consent to the vascular studies

^e^Research samples

^f^CGM will be in place for 10 days after each study visit

^g^Blood pressure pulse wave velocity, central arterial pressure, augmentation index

^h^PIS to be given after diagnosis of GDM

^i^At booking, if available

^j^Samples analyzed within 3 weeks before baseline can be used. If unavailable, an HbA1c sample must be collected at baseline

^k^Sample should be produced at approximately 36 weeks

^l^Food diaries will be given to participants to be completed the 4 days prior to follow-up 2. This should occur prior to providing a fecal sample

^m^Collected at each visit from baseline to discharge from hospital of mother and infant

^n^Medications given in labor do not need to be recorded

^o^From the umbilical cord. If *missed*, a heel prick from the newborn will be collected within 72 h of birth with consent

^p^Retrieved from medical notes

^q^Apgar score: 5 min post birth

^r^HbA1c samples will be collected at the local GP as per routine care, and results requested

^s^Fasted and 2 h post-prandial glucose taken from the OGTT appointment

^t^Optional assessment: to be done where a validated set of clinical scales are available

^u^Only if the visit is face to face

### Sample size {14}

A study size of 158 participants will provide sufficient statistical power to detect a 6% reduction of 0.304 mmol/L in maternal fasting glucose at 36 weeks, while allowing for a 18% withdrawal rate (complete data on 130 women).

Based on data from the UPBEAT study [54], we anticipate a mean of 5.06 mmol/L, reduced to 4.756 in the intervention group (6% reduction), SD 0.6 mmol/L, correlation between repeated measures 0.46. Complete data on 130 subjects (65 per arm) will give approximately 90% power to detect a difference in maternal fasting glucose at 36 weeks of 6% (from 5.06 to 4.75 mmol/L).

The 6% reduction is based on a previous study that reported differences with UDCA treatment for nonalcoholic fatty liver disease (NAFLD) in a population of similar body mass index and age to our study group [55]. This difference in glucose concentration is considered clinically relevant as it is equivalent to the difference in glucose categories between which differences in LGA, primary caesarean section, cord blood serum C-peptide level > 90th centile, and clinical neonatal hypoglycemia were evident in the HAPO study [1].

### Recruitment {15}

NHS Trusts who expressed interest in participating in the trial were asked to complete a feasibility form prior to being included, to enable the trial team to assess whether they would be able to meet recruitment targets. The assessment included consideration of how many women with GDM were cared for by the site, and the clinical pathways followed. Due to the COVID-19 pandemic, this was particularly important, as many Trusts reduced the number of face-to-face appointments in favor of virtual appointments. In addition, some Trusts changed their diagnostic criteria for GDM, replacing the OGTT with alternative means of diagnosis. By ensuring that each Trust answered the feasibility questions before being accepted, the likelihood of successful recruitment was increased.

## Assignment of interventions: allocation

### Sequence generation {16a}

Subjects are randomized by remote computerized web-based allocation, provided by MedSciNet^Ltd^. The groups are balanced by BMI category (Normal/Overweight/Obese), previous history of GDM, severity of diagnosis (diagnostic fasting glucose ≥ 6.3 or ≤ 6.2 mmol/L), and by center using a minimization process. Regular checks during the recruitment phase will be carried out to confirm that the minimization procedure has been applied correctly.

### Concealment mechanism {16b}

The use of a web-based allocation system and the minimization process will ensure that the allocation sequence is concealed until the intervention is assigned.

### Implementation {16c}

Participants will be enrolled by the local consenting clinician or research midwives. Eligibility will be confirmed by a clinician on the eCRF, and baseline data will be entered by the local research midwife. Once completed, the research midwife will select “Randomize” on the eCRF to reveal the allocated group. The research midwife is responsible for communicating the allocated group to the participant, their clinician, and the clinical trials pharmacy, who will dispense the IMP.

## Assignment of interventions: blinding

### Who will be blinded {17a}

Due to the difference in pill size and dosage, this is an open-label study; therefore, the participant and clinical and trial teams will not be blinded to allocation. The Postdoctoral Research Associate responsible for the analysis of the samples will remain blinded to participant allocation; however, bile acid concentrations will reveal allocation and therefore these will be the last assays undertaken.

### Procedure for unblinding if needed {17b}

Not applicable, as the intervention is not blinded.

## Data collection and management

### Plans for assessment and collection of outcomes {18a}

#### Primary outcome

##### Maternal fasting blood glucose at 36 weeks’ gestation

Participants will fast for at least 3 h before blood collection by venipuncture by trained members of the research team. The samples will be processed in each hospital’s accredited local central laboratory, with results reported to the participant’s clinical record via each Trust’s electronic reporting system. The electronic system will be the source data from which the result is taken, and then entered onto the eCRF by the local research team.

#### Secondary outcomes

##### To evaluate the impact of the treatments on maternal and neonatal lipid metabolism

Maternal lipid metabolism will be assessed from blood samples at follow-up 2, including blood triglyceride, total cholesterol, calculated LDL cholesterol, HDL cholesterol, and free fatty acid concentrations. The lipid profile will be processed in each Trust’s accredited clinical laboratory. The free fatty acid sample will be processed and stored in the research laboratories by trained staff, following the GUARD laboratory manual. This sample will then be analyzed be the trial team’s Postdoctoral Research Associate.

Neonatal lipids will be assessed in umbilical cord blood, analyzing C-peptide, triglyceride, total cholesterol, calculated LDL cholesterol, HDL cholesterol, and free fatty acid. The samples will be taken from the umbilical cord shortly after birth. If research staff are not available, instructions will be added to participant’s pregnancy notes asking clinical staff to take these bloods, following the same procedure that staff are trained in to take clinically indicated umbilical cord bloods. In the event that these bloods are taken “out of hours,” they can be kept in a fridge up to 72 h while maintaining stability. Two blood spots from the umbilical cord will also be collected onto a Whatmann card for in-depth analysis of individual neonatal lipids, which will be analyzed at Dr Albert Koulman’s collaborating laboratory, Cambridge. In the scenario that this sample cannot be obtained, consent will be gained to take a blood spot from the newborn within 72 h of birth. This will be taken by trained research staff using a lancet to prick the newborn’s heel.

##### To assess the acceptability of UDCA compared with metformin for women with GDM

The acceptability of treatment will be assessed using two validated questionnaires: the Quality of Life questionnaire (EQ-5D-5L) [45] and the Diabetes Treatment Satisfaction Questionnaire (DTSQs) [46]. The EQ-5D-DL questionnaire is a validated tool that is widely used to evaluate the concept of health-related quality of life [45]. The tool includes two pages, the first assesses five dimensions: mobility, self-care, usual activities, pain/discomfort, and anxiety/depression. Participants are asked to rate each dimension on 5 levels: no problems, slight problems, moderate problems, severe problems, and extreme problems. The second page is a visual analog scale rated 0–100, where the top end is “the best health you can imagine” and the bottom is “the worst health you can imagine”; the participant marks “X” at the point that they feel they are, which is then transcribed into a value. The participant will be asked to complete a paper version of this questionnaire at baseline and at follow-up 2.

The participant will also be asked to complete a paper copy of the DTSQs at follow-up 2. The questionnaire is a validated assessment of satisfaction in diabetes treatment [46], particularly in the assessment of new treatments [56]. It comprises 8 questions, each of which is assessed on a scale of 0–6, which the woman circles. Six questions relate to treatment satisfaction, and the remaining two refer to perceptions of glucose control.

The research midwife will transcribe participants’ answers onto the eCRF. The paper questionnaires will be stored as source data, and will undergo data verification checks to ensure they are transcribed correctly, during trial monitoring visits.

##### To establish whether continuous glucose monitoring gives clinically useful information for the assessment of maternal glycemic control in women with GDM

Participants will have a CGM device inserted by the research midwife at each study visit. The device remains in situ for 10 days each time, and then the participant removes the sensor at home. The participant returns the device at each visit, to allow the research midwife to upload the data. All research midwives involved in the trial are trained in the insertion and management of the device and are provided with a manual for reference. DEXCOM G6 devices are used for all participants, a CGM system that has FDA approval [57].

##### To evaluate vascular responses in each arm (optional element)

A calibrated cuff-based blood pressure instrument, the arteriograph, will be used for vascular studies. Two measurements will be taken over a 10-min period, and the readings will be transmitted via Bluetooth from the device to the TensioMed24 software. All staff using the device will receive training by the trial team and will be provided with a manual for reference.

##### To compare maternal and fetal outcomes that could relate to treatment with UDCA or metformin

1. Glucose metabolism at baseline, follow-up 1, and follow-up 2 assessed by:
  Continuous glucose monitoring (CGM) as detailed above.
  Blood samples taken by the research team via venipuncture for: serum concentrations of 1,5-anhydroglucitol, a novel marker of short-term glycaemia [1, 4] which will be initially processed by the research laboratory team and then analyzed at Neil Dalton’s collaborating laboratory in St Thomas’ Hospital; HbA1c concentration (except at fFollow-uUp 1), a conventional marker of medium-term glycaemia that will be processed in each participating Trust’s clinical laboratory.
2. Biochemical analysis of maternal blood for liver function tests at follow-up 2 (ALT, bilirubin, ALP), bile acids, C-reactive protein (including highly sensitive analyses) that will be processed in the clinical laboratories.
3. Proportion of women requiring insulin treatment (time until insulin treatment and maximum daily dose of insulin required). This information will be taken from participants’ medical records.
4. Maternal gestational weight change at 36 weeks compared to weight at first trimester antenatal visit, measured using a set of validated scales
5. Blood pressure and will be measured in triplicate at each visit for participants who are not partaking in the optional vascular element, by trained staff using a validated device.
6. The effect of UDCA/metformin on the maternal gut microbiome will be evaluated through an optional stool sample at 36 weeks gestation. Gut microbiota will be determined in the stool samples by 16S rRNA sequencing. Fecal bile acid profiles will be obtained using UPLC MS/MS, and short-chain fatty acids will be quantified by gas-liquid chromatography (GLC).
7. A sample will be collected from the neonates first meconium stool. This will be stored for future analysis to assess fetal exposure to UDCA/metformin.

The following birth outcomes will be obtained from participants’ medical records, and entered onto the eCRF by the local research staff:

1. Mode of birth (rates of caesarean section (CS), (elective & emergency), instrumental vaginal birth, and spontaneous vaginal delivery (SVD)).
2. Gestational age at birth.
3. Apgar scores @ 5 min post birth
4. Occurrence of shoulder dystocia.
5. Estimated blood loss at time of birth
6. Infant birth weight and a customized birth weight percentile calculated using the GROW centile calculator [49], to assess the proportion of babies born large for gestational age (LGA), proportion of babies born small for gestational age (SGA).
7. Neonatal morbidity (treatment for neonatal hypoglycemia, neonatal jaundice, respiratory distress or birth trauma).
8. Neonatal intensive care and special care unit admission (number of inpatient nights).
9. Stillbirth and neonatal death.

### Plans to promote participant retention and complete follow-up {18b}

The research team will contact participants prior to follow-up visits to confirm attendance, and prior to follow-up 2 participants will be reminded that they must fast for the visit. In the event a participant is unable to attend, for example due to isolation requirements as a result of COVID-19, the visit will be re-arranged for as near to the window as possible. This will be documented as a protocol deviation and will be recorded on the eCRF.

For participants who deviate from the intervention, this will be captured in the eCRF. If participants are on an alternative medication this will be documented under concomitant medications. If a participant has withdrawn from the intervention only, the follow-up data will continue to be collected as per the protocol.

### Data management {19}

Data is managed as per the Data Management Plan (DMP), which identifies and defines the study personnel and roles involved with decision making, data collection, and data handling. The DMP contains the detailed working practices related to each aspect of data management that are sufficient to allow procedures to continue to be followed in the absence of the person usually performing those responsibilities.

#### Data forms and data entry

Data will be recorded directly onto eCRFs on a database designed by members of the study research team and hosted by MedSciNet UK Limited. MedSciNet is an internet-based Electronic Data Capture (EDC) system that is fully validated and secure. Data will be inputted by trial sites’ staff, as authorized by the Principal Investigator according to the delegation log. The trial site staff will create database records for every consented participant on the eCRF. A minimal amount of fully anonymized data will be collected for patients who are approached for the trial but do not consent, to ensure reasons for decline are captured.

Data points on the eCRF contain validation points to ensure that the inclusion criteria are being adhered to; for example, if participants are not within the correct gestational age range, it will not be possible to save and continue. Other data points will trigger an alert that the data entered are outside the normal range, for example a very abnormal blood result, which will prompt staff to check their entry.

Each user will be trained on the eCRF system prior to being granted access to the live database. They will each have a personal account that is password-protected, and different levels of access dependent upon their roles and responsibilities within the trial.

To protect the security of data in the database, site user accounts are issued only when training requirements are met and roles are defined on the signed delegation log.

Each site user will have access to the Trial Handbook, which provides detailed instructions to assist site personnel when entering data.

#### Data queries

Data validation checks provide the first quality control (QC) step, immediately after an eCRF page is saved as complete. These checks ensure the completeness, plausibility, and consistency of manually entered trial data. Programmed edit checks run online at the time of saving each screen/page. Subject data that violates a validation rule will automatically generate a data query, which is instantly visible to the user in the eCRF. Queries can be answered immediately and, if necessary, the data in the eCRF can be changed by the user directly in the online query form.

The Clinical Trials Manager (CTM), Lead Research Midwife (RM), and the Clinical Research Associate (CRA) from the trial team will perform discrepancy management and issue additional queries on any discrepant data or where further clarification is required in relation to system queries. The trial site staff will answer the queries raised and update the eCRF data when appropriate, submitting responses through the eCRF system.

Once the eCRF forms/screens are declared clean and locked (by the trial team members, as delegated by the CI), the Principal Investigator (PI) must complete the signature panel associated with each form/screen.

As part of the ongoing quality control process, the CTM and CRA will monitor the type and number of queries experienced across sites on an ongoing basis. A regularly updated frequently asked questions document will also be circulated among participating sites.

#### Security and storage

All source documents, the eCRF, and data export are kept within secured locations and comply with the Data Protection Act 2018 and GDPR. The data are stored in Guy’s and St Thomas’ Biomedical Research Centre eCRF servers and meet all MHRA requirements for CTIMP data storage. Only the server administrator has access to this server and the core database, via remote connection. The back-up process is twofold: every 24 h, the database is backed up to the server and every 7 days the entire server is backed up to an archival tape system.

All hard copies of source data worksheets and Investigators Site Files (ISF) will be kept in a locked office within each trial site.

All trial data will be stored in line with the Medicines for Human Use (Clinical Trials) Amended Regulations 2006 and the Data Protection Act 2018 and archived in line with the Medicines for Human Use (Clinical Trials) Amended Regulations 2006 as defined in the Kings Health Partners Clinical Trials Office Archiving SOP, for at least 25 years.

Participants will be asked to provide their email addresses if they wish to be informed of study results and to be contacted in the future. These will be collected in the ISF and a copy will be given to the sponsor at intervals or at the end of the trial.

### Confidentiality {27}

Only local site staff will have access to pre-screening, screening, and participant ID logs detailing identifiable data. Where this information is collected electronically, it will be stored on a password-protected document. If this is collected on a paper log, then this will be stored securely in a locked location.

All data stored on the database is pseudo-anonymized and data entry personnel will ensure that patient identifiable data are not included in the text fields of the data entry screens. The participants will be identified in the study database by a unique participant ID number.

Participants’ postcodes will be collected and entered into the eCRF; however, they will not be stored; the eCRF will match the postcode with the Census area (LSOA), which will be saved and displayed in the eCRF.

### Plans for collection, laboratory evaluation, and storage of biological specimens for genetic or molecular analysis in this trial/future use {33}

All samples collected during the trial will be labelled with the participant’s trial ID and will not contain any identifiable data. After arrival at the local research laboratory at each site, the samples will have unique barcodes applied, which will be scanned directly into the participant’s entry on the eCRF. All samples will be stored in freezers at − 80 °C and at the end of the trial will be transferred to King’s College London for analyses. Participants have the option of consenting to their samples being stored for future research. For those that have agreed to this, once the trial is completed their samples will be stored in an HTA-compliant laboratory. The samples of those who have not consented to this element will be destroyed in accordance with HTA rules.

Maternal blood samples will be collected from participants by delegated research staff. Neonatal cord blood will be collected by appropriately trained clinical staff. Upon collection, the samples will be transported to each site’s research laboratory, where they will be processed, aliquoted, and stored by trained staff who will follow the Laboratory Manual.

Neonatal meconium will be collected by clinical staff and transported to the research laboratory where it will be stored at − 80 °C.

Participants who consent to the optional stool sample will produce the sample at home and store in their own freezer, alongside ice packs which will be used for transport. The participant will then contact their local research midwife to arrange a courier, who will transport the sample to the site’s research laboratory. The sample will be barcoded and stored immediately at − 80 °C. All stool samples will be analyzed at King’s College London for bacterial rRNA extraction and 16S sequencing.

The neonatal blood spots will be stored with the aim to analyze lipidomic profiles, which will be undertaken at Albert Koulman’s laboratory in Cambridge.

More detailed information regarding sample analysis can be seen in the Laboratory Manual (supplementary file 1).

## Statistical methods

### Statistical methods for primary and secondary outcomes {20a}

The main analysis will follow the intention to treat (ITT) principle, using all available data on randomized women, according to the intended treatment option. Should there be a large number of women (over 20%) not following the randomized treatment, a per protocol (PP) dataset limited to women following the intended treatment will also be established and a secondary PP analysis will be conducted.

All comparisons by treatment group will be adjusted for BMI, previous history of GDM, disease severity (baseline fasting glucose ≤ 6.2 or ≥ 6.3), and center. Data derived from the CGM will be analyzed at 36 weeks. To increase the power and accuracy of comparisons, multiple regression will adjust for the baseline randomization measurements when examining the differences caused by the randomized treatment.

UDCA will be declared non-inferior to metformin if metformin does not have a significant advantage, and the largest plausible advantage (by 95% confidence interval) is less than 0.28 mmol/L. If neither treatment shows a significant advantage, and the difference and CI are less than 0.28 mmol/L, the treatments will be regarded as equivalent.

Table 3 summarizes the statistical methods used to report the primary outcome and powered secondary outcomes, and Table 4 illustrates the additional unpowered outcomes.

**Table 3:** Comparison of primary and powered secondary outcomes by randomized treatment group

	Treatment	Control	Comparison^a^ (difference or risk ratio) with 95% CI	Significance^a^
**Primary endpoint**				
Fasting glucose^b^	*N* Mean (SD)	*N* Mean (SD)	Difference (95% CI)	*P* = 0.xxx
**Powered secondary endpoints (maternal)**				
Fasting total cholesterol	*N* Mean (SD)	*N* Mean (SD)	Difference (95% CI)	*P* = 0.xxx
Fasting LDL cholesterol	*N* Mean (SD)	*N* Mean (SD)	Difference (95% CI)	*P* = 0.xxx
**Powered secondary endpoint (fetal)**				
Free fatty acids (cord blood)	*N* Mean (SD)	*N* Mean (SD)	Difference (95% CI)	*P* = 0.xxx
Triglycerides (cord blood)	*N* Geometric mean (SD)	*N* Geometric mean (SD)	Difference (95% CI)	*P* = 0.xxx

^a^ Significance tests for pre-planned primary and main secondary outcomes only

^b^ Comparisons are adjusted for minimization variables (BMI Previous GDM and baseline fasting glucose), and baseline measurements where available

**Table 4:** Comparison of additional unpowered outcomes by randomized treatment group

	Treatment	Control	Comparison^a^ (difference or risk ratio) with 95% CI
Quality of life	*N* Mean (SD)	*N* Mean (SD)	Difference (95% CI)
Treatment satisfaction	*N* Mean (SD)	*N* Mean (SD)	RR (95% CI)
**Blood glucose control**			
CGM percentage time within target	*N* Mean (SD)	*N* Mean (SD)	Difference (95% CI)
CGM percentage time above target	*N* Mean (SD)	*N* Mean (SD)	Difference (95% CI)
CGM percentage time below target	*N* Mean (SD)	*N* Mean (SD)	Difference (95% CI)
CGM glucose mean (mmol/L)	*N* Mean (SD)	*N* Mean (SD)	Difference (95% CI)
Serum concentrations of 1,5-anhydroglucitol (μmol/L)	*N* Mean (SD)	*N* Mean (SD)	Difference (95% CI)
HbA1c concentration mmol/L	*N* Mean (SD)	*N* Mean (SD)	Difference (95% CI)
**Lipid metabolism**			
HDL (mmol/L)	*N* Mean (SD)	*N* Mean (SD)	Difference (95% CI)
Triglycerides (mmol/L)	*N* Mean (SD)	*N* Mean (SD)	Difference (95% CI)
**Liver function test**			
ALT (IU/L)	*N* Mean (SD)	*N* Mean (SD)	Difference (95% CI)
Bile acids (μmol/L)	*N* Mean (SD)	*N* Mean (SD)	Difference (95% CI)
C-reactive protein (mg/L)	*N* Mean (SD)	*N* Mean (SD)	Difference (95% CI)
Proportion of women requiring insulin	x/n (%)	x/n (%)	RR (95% CI)
- Time until treatment			
- Maximum daily dose required			
Maternal gestational weight change at 36 weeks compared with first trimester screening visit	*N* Mean (SD)	*N* Mean (SD)	Difference (95% CI)
**Vascular responses**			
Maternal pulse wave velocity (PWV) (m/s)	*N* Mean (SD)	*N* Mean (SD)	Difference (95% CI)
Systolic blood pressure (mmHg)	*N* Mean (SD)	*N* Mean (SD)	Difference (95% CI)
Diastolic blood pressure (mmHg)	*N* Mean (SD)	*N* Mean (SD)	Difference (95% CI)
Central arterial pressure (cP) (mmHg)	*N* Mean (SD)	*N* Mean (SD)	Difference (95% CI)
Augmentation index (AIx) (%)	*N* Mean (SD)	*N* Mean (SD)	Difference (95% CI)
**Birth outcomes**			
Obstetric anal sphincter injury	x/n (%)	x/n (%)	RR (95% CI)
Estimated blood loss at birth	*N* Mean (SD)	*N* Mean (SD)	Difference (95% CI)
Mode of birth			
- Elective caesarean section	x/n (%)	x/n (%)	RR (95% CI)
- Emergency caesarean section	x/n (%)	x/n (%)	RR (95% CI)
- Assisted vaginal birth	x/n (%)	x/n (%)	RR (95% CI)
- Spontaneous vaginal delivery	x/n (%)	x/n (%)	[Reference group]
- Rate of caesarean section	x/n (%)	x/n (%)	RR (95% CI)
Preterm birth < 37 weeks	x/n (%)	x/n (%)	RR (95% CI)
Preterm birth < 34 weeks	x/n (%)	x/n (%)	RR (95% CI)
**NEONATAL**			
Apgar score at 5 min			
Occurrence of shoulder dystocia	x/n (%)	x/n (%)	RR (95% CI)
**Cord blood:**			
C-peptide (Pg/mL)	*N* Geometric mean (SD)	*N* Geometric mean (SD)	Ratio of means
Calculated LDL cholesterol (mmol/L)	*N* Geometric mean (SD)	*N* Geometric mean (SD)	Ratio of means
HDL cholesterol (mmol/L)	*N* Mean (SD)	*N* Mean (SD)	Difference (95% CI)
Free fatty acids(mmol/L)	*N* Geometric mean (SD)	*N* Geometric mean (SD)	Ratio of means
**Infant birth weight**			
Standard deviation scores	*N* Mean (SD)	*N* Mean (SD)	Difference (95% CI)
- Customized birth weight percentile	*N* Mean (SD)	*N* Mean (SD)	Difference (95% CI)
- Proportion of large for gestational age (LGA)	x/n (%)	x/n (%)	RR (95% CI)
- Proportion of small for gestational age (SGA)	x/n (%)	x/n (%)	RR (95% CI)
**Neonatal morbidity**			
Hypoglycemia	x/n (%)	x/n (%)	RR (95% CI)
Jaundice	x/n (%)	x/n (%)	RR (95% CI)
Respiratory distress	x/n (%)	x/n (%)	RR (95% CI)
Birth trauma	x/n (%)	x/n (%)	RR (95% CI)
Neonatal unit admission	x/n (%)	x/n (%)	RR (95% CI)
Duration of hospital stay	*N* Mean (SD)	*N* Mean (SD)	Difference (95% CI)
Stillbirth	x/n (%)	x/n (%)	RR (95% CI)

^a^ Comparisons are adjusted for minimization variables (BMI Previous GDM and baseline fasting glucose), and baseline measurements where available

#### Analysis of primary outcome

Multiple regression will be used to compare concentrations of fasting glucose at 36 weeks between treatment groups, with adjustments for baseline fasting glucose, BMI, and previous history of GDM.

#### Analysis of powered and unpowered secondary outcomes

Percentages and risk ratios for binary outcomes and differences in the mean value for continuous measures will be calculated, except when log transformations are needed, when the ratio of the geometric means will be used. Power calculations have been performed for the following secondary outcomes: maternal fasting total cholesterol, maternal fasting LDL cholesterol, fetal cord blood free fatty acids, and fetal triglycerides.

### Interim analyses {21b}

The Data Monitoring Committee (DMC) will review outcomes shortly after 40 (25%) participants will have given birth. Interim analysis will be performed and reported to the DMC, including rates of adverse pregnancy outcomes and to identify causes for participant withdrawal or non-compliance. Serious adverse events will be reviewed.

The DMC may recommend stopping the trial for several reasons:

- Safety—if the evidence from any serious unexpected adverse event (SUAE) or the primary outcome suggests that the randomized treatment is doing more harm than good.
- Futility—if the recruitment and retention rates suggest that there is little or no chance of the trial answering the research question in the available time.
- Efficacy—if the interim results are so strongly favorable to one treatment, or the other, that it is no longer ethical to randomize patients. This stopping rule will be based on the Haybittle-Peto principle [58] that the trial should continue except in the face of overwhelming evidence (*P* < 0.0001), sufficient to make a recommendation affecting all future pregnant women.

After each meeting the chair of the DMC will write a letter to the Chair of the Trial Steering Committee advising whether it is appropriate for the study to continue.

### Methods for additional analyses (e.g., subgroup analyses) {20b}

In this study, we do not expect to have sufficient numbers to allow for useful subgroup analysis.

### Methods in analysis to handle protocol non-adherence and any statistical methods to handle missing data {20c}

#### Missing data

We will follow a 4-point framework for dealing with incomplete observations, which will allow the correct method to be chosen and subsequently implemented [59]. This framework highlights the importance of using plausible assumptions with regard to the nature of the missing data.

1. Attempt to follow up all randomized participants, even if they withdraw from allocated treatment.
2. Perform a main analysis of all observed data that is valid under a plausible assumption about the missing data. Specifically, we will assume data is missing at random (MAR). Under this assumption, imbalances between treatment groups due to dropout can be corrected by appropriate multiple regression models.
3. Perform sensitivity analyses to explore the effect of departures from the assumption made in the main analysis. The missing not at random (MNAR) analysis will use the method of White and colleagues [59].
4. Account for all randomized participants, at least in the sensitivity analyses.

Furthermore, we will check whether there is an imbalance or if the percentage of missing data within each treatment allocation is similar.

### Plans to give access to the full protocol, participant-level data, and statistical code {31c}

Individual participant data will be deposited in the KORDS depository https://www.kcl.ac.uk/researchsupport/managing/preserve; data will be made freely available to collaborators on application to the trial CI or a nominated deputy from the research team.

We do not anticipate that the trial will result in creation of novel statistical code; however, full statistical methodology will be provided in the reporting manuscript, including any non-conventional code or appropriate references.

## Oversight and monitoring

### Composition of the coordinating center and trial steering committee {5d}

The Clinical Trial Manager (CTM) along with the Chief Investigator (CI) and Clinical Research Associate (CRA) will be responsible, on a day-to-day basis, for overseeing and coordinating the work of the multi-disciplinary trial team.

### Trial Management Group (TMG)

The TMG will be chaired by the CTM and will include the CI, selected co-investigators (or delegated individuals), the RM, and a research matron, statistician, and CRA. For selected meetings, the TMG may additionally include the Trial Pharmacist, representatives from the Kings Health Partners Clinical Trials Office (KHP-CTO) and the Trial Sponsors as required. This group will have responsibility for the day-to-day operational management of the trial. Regular meetings of the TMG will be held to discuss and monitor trial progress and solve problems.

### Trial Steering Committee (TSC)

The TSC was established prior to the start of the study, with a mix of independent and study team members, and meets at least annually. The TSC is an executive committee, responsible for the overall supervision on behalf of the Sponsor and the Funder, ensuring the trial is conducted in accordance with the rigorous standards set out in the UK Policy Framework for Health and Social Care Research and Guidelines for Good Clinical Practice, safeguarding the rights, safety, and wellbeing of participants. The TSC consists of an independent chair, the CI, the statistician, and independent members consisting of an obstetrician, two diabetologists, a midwife, and two patient and public involvement members. The TSC discusses recommendations raised by the DMC. The members agreed upon a charter listing the detailed Terms of Reference and frequency of meetings.

Responsibilities:

- Reviewing selection/recruitment/retention of participants and their management.
- Determine if amendments to the protocol or changes to study conduct are required and deciding on changes to these and to study conduct in general. Any changes to trial documentation or conduct must be notified to the TSC.
- Assessing the impact and relevance of external evidence.
- Assessing integrity and completeness of data collected.
- Monitoring the overall conduct of the trial, ensuring that it follows the standards set out in the guidelines of GCP, assessing the safety and efficacy of the interventions, recruitment figures, and completion of trial assessments.
- Reviewing, commenting, and making decisions on extension requests.
- Reviewing the recommendations of the DMC and suggesting appropriate action to the TMG.
- Monitoring the progress of trial and deciding on appropriate action in order to maximize the chances of completing it within the agreed timelines.
- Considering new information relevant to the study, e.g., results from other studies that may have a bearing to the conduct of the study and deciding on appropriate action.
- Endorsing the quarterly report to the funder.
- The TSC may decide on early termination of the trial or modification of the study design in the event of a clear outcome derived from accumulating data or on the basis of information available from other sources or on safety grounds.
- The TSC should be available to provide independent advice as required not just when meetings are scheduled.

#### Data management team

The CTM, with the support of the Clinical Data Manager (CDM) and the CRA, is responsible for the creation and maintenance of the Data Management Plan (DMP) during the course of the trial and will ensure that it is implemented accordingly.

The CI will act as custodian for the trial data and has oversight of the data management processes and should, in discussion with the CTM, delegate where appropriate any ad hoc data management activities.

The CTM, CRA, and RM are responsible for the data cleaning of the study data, and the CRA is responsible for the data monitoring of trial data.

The database will not be finalized, locked, and/or archived unless all project-specific procedures have been approved by the CI.

The CTM is responsible for the day-to-day management processes for the GUARD trial, and in their absence the RM would be the first line of contact.

### Composition of the data monitoring committee, its role and reporting structure {21a}

The Data Monitoring Committee has been established and is independent from the Sponsors and trial investigators. It comprises two fully independent clinicians and an independent statistician. The DMC’s responsibility is to safeguard the interests of the trial participants and advise the TSC, to protect the validity and credibility of the trial. During the recruitment period, reports will be provided to the DMC as per charter, which will include information on the adverse events (AEs) reported, recruitment, along with any other data that the committee may request. The DMC does not make decisions about the trial, but rather makes recommendations to the TSC.

Following a report from the DMC, the TSC will decide what actions, if any, are required. The DMC will meet approximately every 6 months, but the exact frequency will be determined by recruitment levels. The Trial Manager will liaise with the DMC Chair regularly to provide recruitment updates.

### Adverse event reporting and harms {22}

Adverse event data will be collected at each visit from baseline to discharge from hospital of mother and infant. All adverse events will be recorded in the medical notes.

All serious adverse events (SAEs), serious adverse reactions (SARs), and suspected unexpected serious adverse reactions (SUSARs) (except those specified in the protocol as not requiring reporting) will be reported immediately (and certainly no later than 24 h) by the investigator to the KHP-CTO and CI for review in accordance with the current Pharmacovigilance Policy, and subsequently recorded in the eCRF. All SAEs will be reported using MedDRA coding, in liaison with the study CRA.

SAEs, SARs, and SUSARs are defined as per The Medicines for Human Use (Clinical Trials) Regulations 2004 and Amended Regulations 2006 as any adverse event, adverse reaction, or unexpected adverse reaction, respectively, that:

- Results in death (including neonatal),
- Is life-threatening,
- Required hospitalization or prolongation of existing hospitalization,
- Results in persistent or significant disability or incapacity, or
- Consists of a congenital anomaly or birth defect.

The KHP-CTO will report SUSARs to the regulatory authorities including the MHRA, and the CI will report to the relevant Ethics Committee.

The CI and KHP-CTO (on behalf of the co-sponsors) will submit a Development Safety Update Report (DSUR) relating to this trial IMP, to the MHRA and REC annually.

The assignment of the causality will be made by the investigator responsible for the care of the participant. An AE whose causal relationship to the study drug or study procedure is assessed by the investigator as “possibly,” “likely,” or “definitely” is an adverse drug reaction, and if graded as serious and unexpected will be reported as a SUSAR to the competent authority by the sponsor.

Events that are reported as outcomes in the eCRF, or those that are expected in this population or as result of routine care/treatment, will not need to be reported as AEs or SAEs. Those events will only be reported to the sponsor if the investigator believes the event is a result of the GUARD intervention. All unexpected SAR will be reported.

### Frequency and plans for auditing trial conduct {23}

Monitoring of this trial will be performed to ensure compliance with Good Clinical Practice, and scientific integrity will be managed, and oversight retained by the KHP-CTO Quality Team. A monitoring plan was developed by the KHP-CTO on the basis of the risk assessment. The KHP-CTO will carry out on-site monitoring to undertake source data verification checks and confirm that records are being appropriately maintained by the PI and pharmacy teams. If on-site visits are not possible, arrangements will be in place for remote monitoring. The site PI will be responsible for ensuring the findings are addressed appropriately. The CTM will ensure relevant findings are discussed with the CI and the report is filed in the Trial Master File (TMF).

### Plans for communicating important protocol amendments to relevant parties (e.g., trial participants, ethical committees) {25}

Any changes to the protocol that would impact the study design, participant safety or eligibility, or any other study procedures, will be submitted to the REC board, the MHRA, and the Research and Development department for approval before implementing.

Participants will be informed of any changes during their involvement in the study, and reconsented with updated ICFs as appropriate.

The clinicaltrials.gov trial registry will be updated accordingly with any substantial amendments that are implemented.

### Dissemination plans {31a}

Ongoing progress of the trial will be disseminated to the wider clinical community through relevant professional newsletters, meetings, and national and international conferences.

The final report to the funder(s) will present detailed results of the trial. The trial will be reported in accordance with the Consolidated Standards of Reporting Trials (CONSORT) guidelines (www.consort-statement.org).

A lay person’s summary of the principal findings of the results will be sent to all participants who have consented to having their email addresses collected for the purpose of being informed of trial results.

Articles will be prepared for relevant professional journals as well as for peer-reviewed scientific journals.

## Discussion

The ongoing challenges presented by the COVID-19 pandemic continue to impact care pathways, and consequentially the delivery of clinical trials. As such, the trial team are continually assessing any potential barriers that may impact successful delivery of the trial, and where improvements can be made without affecting the integrity of the trial, will adjust the protocol accordingly. Any significant changes to the protocol will be submitted as an update to the Trials journal.

## Trial status

Recruitment commenced in July 2021 and is expected to be completed by January 2023. The protocol is v4.0 dated 21 October 2021.

## Supplementary Information

**Additional file 1.**

**Additional file 2.**

## Data Availability

The final data set will be available to members of the trial management team, and anonymized data will be available to research collaborators.

## Abbreviations

AE: Adverse event
AR: Adverse reaction
BD: Twice a day
BMI: Body mass index
BRC: Biomedical Research Centre
CGM: Continuous glucose monitoring
CI: Chief Investigator
CRA: Clinical Research Associate
CS: Caesarean section
CTIMP: Clinical Trial of an Investigational Medicinal Product
CTM: Clinical Trial Manager
CTO: Clinical Trials Office
eCRF / CRF: (Electronic) Case Report Form
FU: Follow-up
GCP: Good Clinical Practice
GDM: Gestational diabetes mellitus
GSTFT: Guy’s and St Thomas’ NHS Foundation Trust
HBA1C: Glycated hemoglobin
HDL: High-density lipoprotein
ICF: Informed consent form
ICH: International Conference on Harmonisation
ICP: Intrahepatic cholestasis of pregnancy
DMC: Independent Data Monitoring Committee
IMP: Investigational Medicinal Product
ISF: Investigator site file
ITT: Intention to treat
QOLQ: Quality of Life Questionnaire
KCP: King’s Health Partners
KCL: King’s College London
LGA: Large gestational age
LSCS: Lower segment Caesarian section
NICE: National Institute for Health and Care Excellence
NICU: Neonatal intensive care unit
NIMP: Non-Investigational Medicinal Product
OGTT: Oral glucose tolerance test
PI: Principal Investigator
PIS: Patient Information Sheet
PMU: Pharmacy Manufacturing Unit
PPI: Patient and Public Involvement
PWV: Maternal pulse wave velocity
RCT: Randomized controlled trial
SAE: Serious adverse event
SAR: Suspected adverse reaction
SMBG: Self-monitoring of blood glucose
SmPC: Summary of Product Characteristics
SUSAR: Suspected unexpected adverse reaction
SVD: Spontaneous vaginal delivery
T2DM: Type 2 diabetes mellitus
TMF: Trial Master File
TSC: Trial Steering Committee
UDCA: Ursodeoxycholic acid
U&E: Urea & electrolytes
