# Supplementary material for: A multi-centered trial investigating gestational treatment with ursodeoxycholic acid compared to metformin to reduce effects of diabetes mellitus (GUARD): a randomized controlled trial protocol
Source: Trials. 2022 Jul 19;23:571. doi: 10.1186/s13063-022-06462-y (PMC9295112; doi:10.1186/s13063-022-06462-y)
Supplement: Supplementary file 1 — Additional file 1. [file 13063_2022_6462_MOESM1_ESM.pdf]

GUARD Laboratory Manual

Randomised controlled trial of **G**estational treatment with **U**rsodeoxycholic **A**cid compared to Metformin to **R**educe effects of **D**iabetes mellitus

Version 3.0, 18/06/2021

Coordinating Centre Contact Information

Address

C/O GUARD Trial Group

Williamson Laboratory, Room 2.32W

2nd Floor, Hodgkin Building Guy's Campus, King's College London, London, SE1 1UL

Chief Investigator

Professor Catherine Williamson

Telephone: 020 7848 6350 / 020 7848 6014

Email: catherine.williamson@kcl.ac.uk

Trial Manager

Noelia Pitrelli

Telephone: 020 7188 7188 ext. 53369

Email: noelia.pitrelli@gstt.nhs.uk

Postdoctoral Research Associate

Dr Alice Mitchell

Telephone: 020 7848 6202

Email: alice.mitchell@kcl.ac.uk

Clinical Research Associate

Eliya Nenkova

Telephone: 077 1866 9698

Email: eliya.nenkova@kcl.ac.uk

When contacting, please include the following information:

- Name, email address, telephone number
- Site details, including site ID number
- Patient trial number (if applicable)

This laboratory manual for the GUARD study was produced by Dr Alice Mitchell, and read and approved by the CI, Professor Catherine Williamson.

ALICE MITCHELL

Date: 23/06/2021

CATHERINE WILLIAMSON

Date: 23/06/2021

Table of Contents

1. Introduction 6
2. List of Abbreviations 7
3. Overview 8
   - 3.1 Sample schedule 8
   - 3.2 Sample collection overview 9
4. Equipment 10
5. Sample collection/processing 10
   - 5.1 FO (grey top) vacutainer collection procedure 11
     - 5.1.1 Supplies required 11
     - 5.1.2 Sample collection 11
     - 5.1.3 Sample shipment to in-hospital laboratory 11
   - 5.2 SST (yellow top) vacutainer collection procedure 12
     - 5.2.1 Supplies required 12
     - 5.2.2 SST serum collection 12
       - 5.2.2.1 SST serum to send to in-hospital laboratory 12
       - 5.2.2.2 Research SST serum 12
   - 5.3 EDTA (purple top) vacutainer collection procedure 13
     - 5.3.1 Supplies required 13
     - 5.3.2 EDTA plasma collection 13
     - 5.3.3 EDTA plasma separation 14
       - 5.3.3.1 EDTA plasma to send to in-hospital laboratory 14
       - 5.3.3.2 Research EDTA plasma 14
   - 5.4 Cord blood collection 15
     - 5.4.1 Supplies required 15
     - 5.4.2 Cord blood collection procedure 15
     - 5.4.3 Neonatal heel prick 16
   - 5.5 GUARD-MEC patient meal and sample collection procedure (ST and QCCH only) 17
6. Faecal sample collection procedure 19
     - 6.1.1 Supplies required 19
     - 6.1.2 Faecal sample collection 19
     - 6.1.3 Faecal sample storage and logging 20
     - 6.1.4 At-home faecal sample collection instructions 20
7. Meconium collection 21
     - 7.1.1 Supplies required 21
     - 7.1.2 Meconium sample processing 21

7.1.3 Meconium sample storage and logging 21
8. Sample Logs 21
9. Storage of serum/plasma 22
9.1 Important notes 22
9.2 Cryo box layout and tube placement for serum/plasma 22
9.3 Logging Sample Barcodes 22
9.4 Freezing blood samples 24
10. Missing or illegible labels 25
11. Sample transfer to CI's laboratory 25
11.1 Arranging a courier collection 25
11.2 Sample packaging 25
11.3 Documentation 25
11.3.1 Shipment labels 25
11.3.2 Shipment logs 26
11.3.3 Sample logs for shipping 26
11.3.4 Receiving samples 26
12. Freezer monitoring 27
12.1 Freezer information required to be recorded 27
12.2 Reporting a temperature excursion 27
13. Sample Disposal 28
14. End of study 28
14.1 Shipping final samples 28
14.2 Shipping temperature logs once all samples have been sent 28
Stool Collection Instructions 29
GUARD Laboratory Manual Training Log 33
GUARD - Maternal Blood Sample Collection Form 34
GUARD – Meconium Collection Form 35
GUARD - Maternal Blood Sample Log 36
GUARD-MEC Blood Sample Log ..... **Error! Bookmark not defined.**
GUARD - Cord Blood Sample Log 37
GUARD - Blood Spot Sample Log 38
GUARD – Maternal Stool Sample Log 39
GUARD – Meconium Sample Log 40
GUARD –Box Shipment Log 41
GUARD Temperature Excursion Log 42
GUARD Sample Disposal Form 43

1. Introduction

This manual contains general information about sample handling procedures as well as storage and shipping for the Clinical Trial GUARD and its sub-study GUARD MEC. It is applicable to all trial staff involved in the collection, handling, processing and transfer of samples.

Sites should follow local standard operating procedures (SOPs) regarding collection of blood, centrifuge preparation and maintenance and any other procedures related to research sampling that are not included in this manual.

PLEASE READ THIS LABORATORY MANUAL CAREFULLY TO ENSURE PROPER HANDLING OF THE SPECIMENS.

Complete the form **(GUARD Laboratory Manual Training Log**) to confirm you understand and agree to follow the procedures detailed.

THANK YOU

2. List of Abbreviations

CRP	C-reactive protein
EDTA	Ethylenediaminetetraacetic acid
FO	Fluoride/Oxalate
GUARD-MEC	GUARD Mechanistic Sub-study
HbA1c	Glycated haemoglobin
HTA	Human tissue act 2004
ISF	Investigator site file
LFTs	Liver function tests
NT	Nottingham Hospital
QCCH	Queen Charlotte's and Chelsea Hospital
SST	Serum separator tube
ST	St Thomas' Hospital
U&E	Urea and Electrolytes

3. Overview

Trial sites

Sites which are enrolling patients and collecting samples for GUARD are:

- Queen Charlotte's and Chelsea Hospital (QCCH)
- St. Thomas' (ST) hospital
- Nottingham (NT) hospital

Only QCCH and ST will be collecting GUARD-MEC samples.

3.1 Sample schedule

This table lists a summary of sample type and in-house laboratory analysis or storage required at the different time points in the study after patient enrolment.

Where indicated, each site will send the appropriate blood collection tubes to the in-house laboratory for direct analysis. Otherwise, indicated samples will be stored at -80°C and shipped to the CI's laboratory for further analysis.

Visit time points description:

1. Baseline – 27-31 weeks' gestation
2. Follow-up 1 – 32±1 weeks' gestation
3. Follow-up 2 – 36±1 weeks' gestation (minimum 3 hour fast required – food diary should also be completed in the preceding 4 days)
4. Birth

3.2 Sample collection overview

The table below details the samples required for each time point. Further details on sample collection can be found in the relevant sections.

Gestation	Sample destination	Number and type of vacutainer samples required	Clinical test(s) required, or storage	Other sample collections (storage only)
**Baseline**				
27^{+0}-31^{+6}	**Clinical**	1 x 6mL EDTA (purple top)	HbA1c (only take this sample if participant has not had HbA1c measured in the past 3 weeks)	
		1 x 3.5mL SST (yellow top)	LFTs, U&E, CRP	
		1 x 3.5mL SST (yellow top)	Bile acids	
	**Research**	1 x 6mL EDTA (purple top) – add inhibitors	Storage	
**Follow Up 1**				
$32^{+0} \pm 1$	**Research**	1 x 6mL EDTA (purple top) – add inhibitors	Storage	
**Follow Up 2**				
$36^{+0} \pm 1$	**Clinical**	1 x 4mL FO (grey top)	Glucose	
		1 x 6mL EDTA (purple top)	HbA1c (only take this sample if participant has not had HbA1c measured in the Follow Up 2 visit window)	
		1 x 3.5mL SST (yellow top)	LFTs, U&E, lipid profile, CRP	
		1 x 3.5mL SST (yellow top)	Bile acids	
	**Research**	1 x 6mL EDTA (purple top) – add inhibitors	Storage	
		1 x 5mL SST (yellow top)	Storage	
				Faecal sample
	Note: for GUARD-MEC participants, see section 5.5 for details			
**Birth**				
NA	**Research**	1-2 x 3.5mL SST (yellow top)	Storage of cord blood (serum and cord blood spots, or neonatal heel prick blood spots)	
				Meconium

Please see the relevant sections in this manual for detailed instructions on how to collect and process each sample.

4. Equipment

Each site will be provided with the following laboratory supplies:

- Venepuncture kit
- Vacutainer collection tubes
- 1mL syringe and 21G needle for addition of protease inhibitor mix to EDTA vacutainers for research
- 5mL syringe and 21G needle for cord blood collection
- Barcoded cryovials and boxes for storage of serum, plasma and meconium
- Barcode scanner
- Faecal sample collection kit
- Sterile scoops
- Specimen boxes for storage of cryovials and faecal/meconium sample tubes
- DPP4 inhibitor in pre-made aliquots
- Whatmann 903 blood spot cards, foil storage pouches and lances

If you find that you are running low on supplies, please contact the Postdoctoral Research Associate via email as soon as possible with a list of the supplies that you need, and they will order more supplies for you.

Equipment **not provided**, but required by each site includes:

- -80°C freezer for storage of samples
- Centrifuge with swinging bucket rotor capable of cooling to +4°C
- Pipettes and corresponding tips (200µL, 1000µL)
- Small ice bucket and ice
- Gloves
- Lab coat

5. Sample collection/processing

Samples to be collected for GUARD are as follows:

- Blood samples:
    - FO (grey top): laboratory analysis of glucose (maternal)
    - SST (yellow top): laboratory analysis of lipids, liver function tests, high-sensitivity C-reactive protein, urea and electrolytes, and total bile acids, or to be stored at -80°C after processing (maternal and cord blood)
    - EDTA (purple top): laboratory analysis of HbA1c, or to be stored at -80°C after processing (maternal)
- Faeces: to be stored at -80°C directly (maternal)
- Meconium: to be stored at -80°C directly (neonatal)
- Blood spot: to be air-dryed in a dark place (48 hours), followed by storage at -80°C in a foil pouch

Samples to be collected for GUARD-MEC are detailed in section 5.5.

Important Notes for all plasma/serum processing:

- Aliquot numbers are a target, not a fixed minimum.
- Volume is a fixed requirement. Do not under-fill aliquot microtubes, as the expectation when they are sent for analysis is that they will contain 250 µl and if there is not enough, it is costly to send additional samples.
- The exception to the “no underfill” rule is if you don’t have enough sample to make any full 250 µl tubes, it would be better to have one tube with as much as possible than no sample at all. If there is less than 250 µl of any aliquot, make a note of the approximate volume on the sample log.
- The final sample can be kept if >250 µl, but this volume variation must be documented on the sample log.

NOTE: Be very careful not to pick up red blood cells when aliquoting. This can be avoided by keeping the pipette above the red blood cell layer and leaving a small amount of serum in the tube.

CHECK: That all aliquot vial caps are the correct colour depending on whether they are serum (red top) or plasma (purple top), and that all lids are secure.

5.1 FO (grey top) vacutainer collection procedure

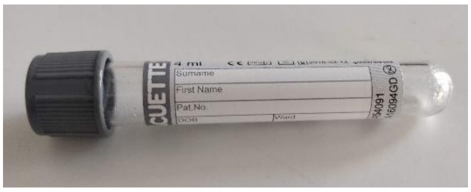

The procedure described below applies to **all sites**.

5.1.1 Supplies required

- 4 ml FO (grey top) vacutainer
- Venepuncture kit
- Gloves (not supplied)
- Lab coat (not supplied)

Please refer to the sample collection overview in section **3.2** for GUARD participants and section **5.5** for GUARD-MEC participants for the number of FO vacutainers to be collected at each visit.

5.1.2 Sample collection

Invert the tube 5-6 times to allow the anti-glycolytic agents to mix with the blood.

5.1.3 Sample shipment to in-hospital laboratory

All FO vacutainer samples will be sent to the in-hospital laboratory. No samples will be stored for further analysis.

Print off label and attach to the sample tube as per local regulations. Send to the in-hospital laboratory as soon as possible (**within 2 hours**) for analysis of **blood glucose**. Samples can remain at room temperature throughout.

Record sample collection on the **GUARD - Maternal Blood Sample Collection Form** and on MedSciNet.

5.2 SST (yellow top) vacutainer collection procedure

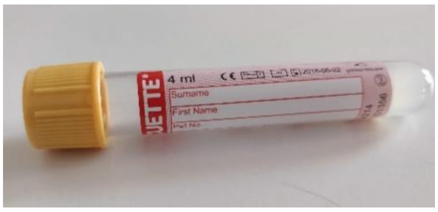

The procedure described below applies to **all sites**.

Different numbers of SST vacutainers will be collected at each time point. Please refer to the sample collection overview in section **3.2** for GUARD participants and section **5.5** for GUARD-MEC participants for the number and size of SST vacutainers needed to be collected at each visit. The in-hospital laboratory tests required are also detailed in these sections.

5.2.1 Supplies required

- 3.5 mL SST (yellow top) vacutainers
- 5 mL SST (yellow top) vacutainers
- Venepuncture kit
- Centrifuge with swing bucket rotor, pre-chilled to 4°C (not supplied)
- Sterile barcoded cryovials and storage box
- 1000 µL pipette and corresponding tips (not supplied)
- Gloves (not supplied)
- Lab coat (not supplied)
- -80°C freezer (not supplied)

5.2.2 SST serum collection

5.2.2.1 SST serum to send to in-hospital laboratory

SST serum tubes will be analysed using the in-hospital laboratory. Print off the label and attach to the sample tube as per local regulations. Send to in-hospital laboratory as soon as possible (**within 2 hours**).

Details of which tests are required at each visit, and the size of the vacutainer, can be found in section **3.2**.

Please note that a separate vacutainer must be used for total bile acids, however the assays for liver function tests, urea and electrolytes, C-reactive protein and lipid profiling can be done using the same vacutainer.

5.2.2.2 Research SST serum

Procedure:

One SST serum tube collected will be taken for storage.

Invert the filled vacutainer 5-6 times to allow it to mix with the clotting agents. Leave the vacutainer to **stand upright for 30 minutes**.

Fill in the **GUARD - Maternal Blood Sample Collection Form** and bring the sample(s) and the form up to the sample processing team. Record sample collection on MedSciNet in the relevant section.

Processing:

Centrifuge the vacutainer at 1100-1300 g for 10 minutes at 4°C.

Use a 1000 µL pipette to transfer **250 µl serum** from the gold-top, SST vacutainer into each of **up to 6** x 0.7 ml cryotubes. Please ensure that each aliquot contains a minimum of 250µL. Seal with red lids. Discard vacutainer containing remaining red blood cells. This process must be completed **within 30 minutes** of centrifugation.

Fill in the **GUARD - Maternal Blood Sample Log** with all the relevant details and freeze the samples as soon as possible. Please see section 6 for details on storage and logging samples into MedSciNet.

5.3 EDTA (purple top) vacutainer collection procedure

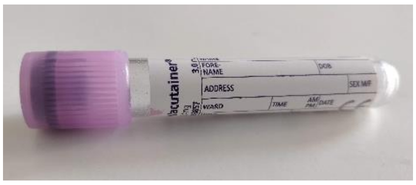

The procedure described below applies to **all** sites.

5.3.1 Supplies required

- 1-2 x 6 mL EDTA (purple top) vacutainer per patient visit
- Venepuncture kit
- 1mL syringe and 21G needle for addition of protease inhibitors to EDTA vacutainers for research
- Complete Protease inhibitor cocktail and DPP4 inhibitor in pre-mixed aliquots
- Centrifuge with swing bucket rotor, pre-chilled to 4°C (not supplied)
- Sterile barcoded cryovials and storage box
- Pipettes (1000 µL) and corresponding tips (not supplied)
- Ice and ice box (not supplied)
- Gloves (not supplied)
- -80°C freezer (not supplied)

5.3.2 EDTA plasma collection

Different samples requiring collection in EDTA vacutainers will be needed at different visits. Please refer to the sample collection overview in section **3.2** for GUARD participants and section **5.5** for GUARD-MEC participants.

For each research sample, make sure that you defrost the correct number of pre-mixed protease inhibitor aliquots before you collect blood samples, and place these aliquots on ice at all times until use. Samples sent for in-hospital analysis do not need protease inhibitors added.

Fill in the **GUARD - Maternal Blood Sample Collection Form** and log the collection into MedSciNet once all samples have been collected.

5.3.3 EDTA plasma separation

5.3.3.1 EDTA plasma to send to in-hospital laboratory

One EDTA plasma tube will be analysed for HbA1c using the in-hospital laboratory. Print off label and attach to the sample tube as per local regulations. Send to in-hospital laboratory as soon as possible (**within 2 hours**).

5.3.3.2 Research EDTA plasma

Procedure:

This applies to **all** EDTA vacutainer samples collected for storage.

Place the vacutainer in a rack on a flat surface and steady if needed with your fingers underneath the rack top to minimise risk of needle stick injury. Ensure you have a sharps bin within reach.

Immediately add the protease inhibitor aliquot DPP4 inhibitor vildagliptin (. Each aliquot should contain approximately 100µL. Using a 1mL syringe and 21G needle, take up the inhibitor aliquot and insert through the top of the EDTA vacutainer tube. Invert the filled vacutainer 5-6 times and **place on ice immediately**. Discard the needle into a sharps bin (do not re-sheath the needle).

NOTE: If a inhibitor aliquot is thawed and not used, please mark the top of the Eppendorf tube with a dot and place it back in the freezer. Freeze-thawing of the protease aliquots may be done twice (two dots marked on the top of the tube). On the third time of taking the aliquot out of the freezer, the contents must be used or discarded to avoid significant degradation of the proteases.

CHECK: That the protease inhibitor mix has been added to the blood sample before delivering the sample to the sample processing team.

Fill in the **GUARD - Maternal Blood Sample Collection Form** and bring the sample(s) on ice and the form up to the sample processing team. Record sample collection on MedSciNet.

Processing:

Centrifuge the vacutainer **within 15 minutes of collection** at 1100-1300 g for 10 minutes at 4°C.

Use a 1000 µL pipette to transfer **250 µL aliquots** of the plasma into each of **up to 6** x 0.7 ml barcoded cryovials. Please ensure that each aliquot contains a minimum of 250µL. Seal with purple lids. Discard vacutainer containing remaining red cells. This process must be completed **within 30 minutes** of centrifugation.

Fill in the **GUARD - Maternal Blood Sample Log** with all the relevant details and freeze the samples as soon as possible. Please see section 6 for details on storage and logging samples into MedSciNet.

5.4 Cord blood collection: vacutainer and blood spot samples

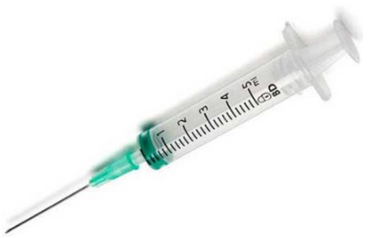

If cord blood collection is needed to check pH as part of clinical care, this must be performed first.

5.4.1 Supplies required

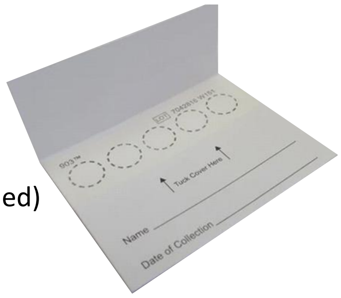

- 2 x 5mL syringe
- 2 x 21G needle
- 2 x 3.5 mL SST (yellow top) vacutainers
- Centrifuge with swing bucket rotor, pre-chilled to 4°C (not supplied)
- 1000 µL pipette and corresponding tips (not supplied)
- Sterile barcoded cryovials and storage box
- Barcoded stickers
- Lancet (for neonatal blood spot collection only)
- Gloves (not supplied)
- Lab coat (not supplied)
- -80°C freezer (not supplied)

5.4.2 Cord blood collection procedure

1. When collecting samples for storage, please use the <u>non-heparin</u> 5 mL syringe supplied and a 21G needle and aspirate gently from the cord artery first.
2. Use the syringe to gently drop blood into one of the dotted circles on the Whatmann 903 blood spot cards until up to 2 of dotted circles are filled, then transfer the remaining sample into a 3.5 mL SST vacutainer and invert the tube 4-5 times to mix the sample. Label the blood spot underneath with "A" for arterial sample and fill in the date and the patient ID in place of "Name". Label the vacutainer with "A", participant ID, date and time, and appointment ("Birth")..
3. Using a separate syringe and needle, repeat this procedure with the cord vein for the blood spot card (fill up to 2 dotted circles) and the vacutainer. Label the blood spot underneath with "V" for venous sample. Label the vacutainer with "V", participant ID, date and time, and appointment ("Birth"). Record sample collection on the Sample Log (section 10.3.3) and on MedSciNet.
4. **NOTE:** *If there is sufficient (> 2 mL) arterial and venous cord blood, then please collect the samples in separate vacutainers. However, if there is not enough cord blood, please pool the arterial and venous samples in the same vacutainer. Please label the samples accordingly ("A" for arterial only, "V" for venous only, or "mixed" where arterial and venous blood have been mixed).*
5. Record sample collection on the **GUARD - Cord Blood Sample Log** and on MedSciNet.
6. Process vacutainer samples according to section 5.2.2.2, ensuring that you record whether the cord blood sample was arterial or venous when logging the aliquot barcodes onto MedSciNet section <u>5.7 Sample collection</u> (see section 6 for instructions), alongside the date and time of collection.
7. Store the blood spot card in a dry, dark place for 24-48 hours without folding the card. Print off matching barcode stickers and attach one to the back of the Whatmann card, and one to the foil storage pouch. After the blood spot has dried for the appropriate time, place the card

into the matched barcoded storage pouch and store in a -80⁰C freezer after logging the sample on MedSciNet (section 6).

5.4.3 Neonatal heel prick

If cord blood is not available, consent should be sought to obtain a neonatal heel prick within 72 hours of birth, with two blood spots to be filled on the Whatmann 903 blood spot cards. Neonatal blood spots should be labelled using "NN" underneath each blood spot and processed exactly as in section 5.4.2. Samples should be logged in MedSciNet using section "Birth".

5.5 GUARD-MEC patient meal and sample collection procedure (ST and QCCH only)

The table below details the timings of venepuncture for women participating in the GUARD-MEC study, the number and type of samples to be taken at each point, and what tests each sample needs to be sent to the in-hospital laboratory for or whether the sample needs to be processed for storage at -80°C. Please refer to each individual section for detailed information on sample collection procedures.

We appreciate that it may be difficult for the participant to come in for exactly 07:50. Therefore, as long as the participant comes in fasted, between 07:30 and 09:30, and the timings for the sample collection schedule are maintained i.e. fasting, 15, 60 and 120 minutes postprandial samples are taken, any variation with starting time should be noted but is acceptable and will not jeopardise the validity of the study.

The breakfast given to the women will consist of 50g fat, 75g carbohydrates, 5g protein, and will be made up from:

- 1 glass of milkshake, consisting of:
    - Sainsbury's Double Cream, 95mL
    - Sainsbury's Soft Scoop Vanilla Ice Cream, 70g
    - Sainsbury's Caster Sugar, 27.5g
- 1 glass of 200mL Sainsbury's Smooth Pure Orange Juice (not from concentrate)

Please note that for GUARD-MEC participants, these samples will be taken instead of those required for Follow-up 2. Please log the samples in the **GUARD - Maternal Blood Sample Collection Form** with the appropriate Visit ID.

		**Number of vacutainer samples required**		
**Time**	**Action**	**FO (grey top) – section 5.1**	**SST (yellow top) – section 5.2**	**EDTA (purple top): ICE required – section 5.3**
**07:50**	*Participant arrives*			
**08:00** (fasting)	Venepuncture	1 x 4 mL (send to laboratory – glucose)	2 x 3.5 mL vacutainers (send to laboratory: 1 x LFTs, U&E, CRP and lipid profile, 1 x total bile acids), plus 1 x 5mL vacutainer for storage	2 x 6 mL (1 x HbA1c, send to laboratory; 1 x storage)
**08:00-08:05**	*Breakfast consumption*			
**08:20** (0h15 postprandial)	Venepuncture	1 x 4 mL (send to laboratory – glucose)	1 x 3.5 mL vacutainer (lipid profile – send to laboratory), 1 x 5 mL vacutainer for storage	1 x 6 mL (storage)
**09:05** (1h00 postprandial)	Venepuncture	1 x 4 mL (send to laboratory – glucose)	1 x 3.5 mL vacutainer (lipid profile – send to laboratory), 1 x 5 mL vacutainer for storage	1 x 6 mL (storage)
**10:05** (2h00 postprandial)	Venepuncture	1 x 4 mL (send to laboratory – glucose)	1 x 3.5 mL vacutainer (lipid profile – send to laboratory), 1 x 5 mL vacutainer for storage	1 x 6 mL (storage)

The samples will be processed by the research team at the time of collection and frozen immediately or sent to the in-hopsital laboratory for clinical testing. Fill in the **GUARD - Maternal Blood Sample Collection Form**, and log the clinical samples taken on the **GUARD - Maternal Blood Sample Collection Form** and on MedSciNet. For each sample stage (fasting = M0, 15 minutes = M15, 1 hour = M1H or 2 hours = M2H), separate the aliquots in a plastic ziplock bag marked with M0 (fasting), M15 (15 minutes post-breakfast), M1H (1 hour post-breakfast), or M2H (2 hours post-breakfast) to ensure they do not get mixed up. Take these research samples all together up to the processing team so that they can scan the barcodes and log them correctly into MedSciNet and log and store the samples.

Logging samples onto MedSciNet:

Fasting samples will be recorded in MedSciNet section 4.2 Sample Collection, and postprandial samples completed in 4.6.1 GUARD MEC Assessments.

6. Faecal sample collection procedure

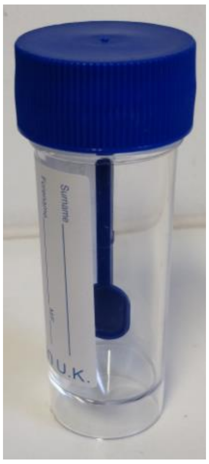

Where possible, we require a faecal sample from the participant at around 36 weeks' gestation. This can be given during their Follow-up 2 appointment, during the study period if they are participating in GUARD-MEC, or at home. Please note that the faecal sample is **optional**. If the woman is unable to give a faecal sample on the day a stool sample collection kit may be provided. If the kit is provided, please relay to the woman that the faecal sample must be stored in their home freezer (at -20°C) until collection.

The participant should contact the site's research midwife once she has collected a faecal sample and give a date and time that suits her for the package to be collected. The research midwife will then contact the coordinating centre who will arrange a courier to collect the package at the earliest possible convenience to the participant. The stool sample will be shipped directly to the CI's laboratory at KCL (please see section **11** for details on shipment protocol). The faecal sample should be shipped on dry ice and stored in a -80°C freezer upon receipt.

6.1.1 Supplies required

- Stool collection kit containing:
  - 1 pair of disposable gloves
  - Hystool flushable stool collection bag with tape pre-attached
  - Sterile specimen pot with attached scoop. **The label on the pot will need to be filled in with the GUARD ID before giving the kit to the participant**
  - Sealed pouch
  - 1 plastic bag for disposal of items
- Food diary to be filled in 4 days prior to stool collection – *this will need to be given to the participant at follow-up 1*
- Gloves
- Lab coat
- -80°C freezer

6.1.2 Faecal sample collection

Stool samples should be passed as per the instruction leaflet using the kit provided (Error! Reference source not found.), either during the appointment period, or at home. Before giving the participant the stool collection kit, mark the collection pot with:

- Sample type: "Stool"
- Study name: "GUARD"
- Participant ID
- Remind the participant to fill in the date they collect their sample on the tube before freezing it in their home freezer if an at-home collection kit is given

No sample processing is required upon receipt – please give the sample to the sample processing team as soon as possible.

6.1.3 Faecal sample storage and logging

Upon receipt of a faecal sample, a frost-proof barcode label should be attached to the pot, the sample scanned into MedSciNet, and it should be **stored immediately** in a -80°C freezer after this. If this is not possible, the sample should be stored on ice for a maximum of 1 hour before being transferred to a -80°C freezer.

Fill in the details on the **GUARD – Maternal Stool Sample Log** and on the relevant section of MedSciNet.

Before freezing, check that the study name, participant ID, sample type and date of the sample are clearly labelled on the collection pot, and that the barcode has been stuck securely to the collection pot.

6.1.4 At-home faecal sample collection instructions

If the participant is unable to give a faecal sample at the Follow-up 2 appointment, they may be given an at-home collection package. The participant should be instructed to follow the instructions carefully (Error! Reference source not found.**)** in the at-home collection package to ensure integrity of the faecal sample.

Before the stool collection kit is given to the participant, each kit should be labelled with their study name (GUARD), participant ID, with space to write the date and time of sample collection, and the date and time of storage. The participant should store the sample in their home freezer and contact the research midwife to arrange courier shipment to the hospital.

7. Meconium collection

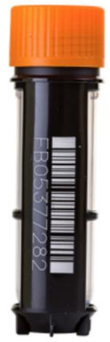

7.1.1 Supplies required

- Sterile scoop
- 5mL Barcoded FluidX tube and storage box
- Gloves
- Lab coat
- -80°C freezer

7.1.2 Meconium sample processing

When a nappy with meconium has been collected by the midwives on the ward, there should be a call up to the Research Midwives' office to tell them a sample has been collected, however, please ensure that you check a minimum of once per day in the fridge on the ward to ensure that no samples are missed.

Each bag with a nappy should come accompanied with the **GUARD – Meconium Collection Form** detailing the participant initials and Study ID, and the date and time of meconium collection. If the time that you pick up the sample is less than 4 hours after the nappy was collected, proceed with the processing instructions below. If the nappy was collected more than 4 hours before you collect it, you must discard the nappy and contents as it has been compromised.

If the sample is less than 4 hours old from collection, scrape the meconium from the nappy using the sterile scoop and place in the 5mL tube. Fill in the Meconium Collection Form and hand this over as soon as possible to the sample processing team.

7.1.3 Meconium sample storage and logging

The meconium sample does not need any further processing and should be **stored immediately** in a -80°C freezer. If this is not possible, the sample should be stored on ice or in a fridge for a maximum of 4 hours from nappy collection (check the Meconium Collection Form) before being transferred to a -80°C freezer. Record collection in the **GUARD – Meconium Sample Log**Error! Reference source not found..

The sample should be logged in MedSciNet against the sample log according to section 9.3 and the file logged in the GUARD Lab folder.

8. Sample Logs

There are four different sample logs which will need completing for each sample that is collected and frozen:

- Maternal Bloods
- Maternal Faeces
- Cord Bloods
- Meconium

These logs must be stored as a record of what samples have been collected and will be required to be scanned when samples are being shipped and the scanned copies added with the shipment and additionally emailed to the receiving site.

9. Storage of serum/plasma

9.1 Important notes

- Serum or plasma samples should be processed and frozen within 1 hour of collection.
- A 2-3 hour window is acceptable if 1 hour is not possible.
- Keep samples on ice until frozen.
- The sample log should be completed including collection, processing and freezing times are documented so that the effects of any delays can be accounted for. The samples will still be of use, but delays must be documented.
- It is essential that all tube barcodes are recorded in the database once the tube has been filled. If MedSciNet is not available, please complete an Excel Proforma and upload to MedSciNet when next possible. *If the barcodes are not recorded, the sample will not be identifiable and will have to be destroyed.*

9.2 Cryo box layout and tube placement for serum/plasma

Columns (1 – 12)

Rows (A – H)

A1	A2	A3	A4	A5	A6	A7	A8	A9	A10	A11	A12
B1	B2	B3	B4	B5	B6	B7	B8	B9	B10	B11	B12
C1	C2	C3	C4	C5	C6	C7	C8	C9	C10	C11	C12
D1	D2	D3	D4	D5	D6	D7	D8	D9	D10	D11	D12
E1	E2	E3	E4	E5	E6	E7	E8	E9	E10	E11	E12
F1	F2	F3	F4	F5	F6	F7	F8	F9	F10	F11	F12
G1	G2	G3	G4	G5	G6	G7	G8	G9	G10	G11	G12
H1	H2	H3	H4	H5	H6	H7	H8	H9	H10	H11	H12

- Use these coordinates to note which sample will be placed in which position.
- Please put samples in along the rows, not vertically.
- Ensure the boxes are used in the correct orientation with A1 in the top left as shown, and that the lid is securely fastened when finished, using the lock symbols as a guide.
- Do not force the lid on, attach it carefully.
- Make a note of the first and last aliquot positions and the box barcode and position on the sample log.

9.3 Logging Sample Barcodes

Sample barcodes will be linked to the appropriate study participant via a MedSciNet database.

Barcodes must be recorded immediately after processing to avoid losing this link between participant and sample.

1. Load website: https://guard.medscinet.com/
2. Log in using your own login credentials (never use another's login or tell anyone yours)
3. Search and select the correct participant ID

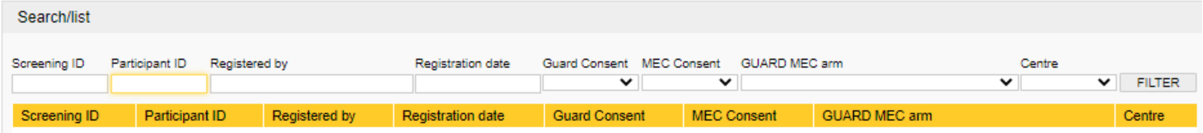

4. Check the sample log to confirm the date and time matches the vacutainers you received.
5. Navigate to the correct "Sample collection" page.

   Research samples will be logged in the following sections:

   Baseline – 2.4 Sample collection

   Follow-up 1 ("FU1") – 3.2 Research sample collection and storage

   Follow-up 2 ("FU2") – 4.2 Sample collection

   GUARD-MEC *where applicable* – 4.6.1 GUARD MEC Assessments

   Birth – 5.7 Sample collection

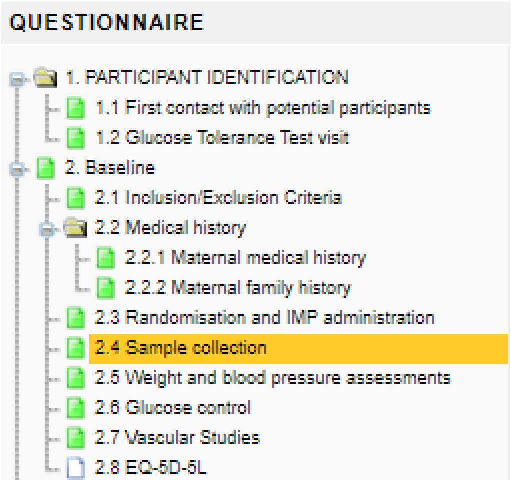

6. Fill in the "Research Samples" section according to the vacutainer type you are processing

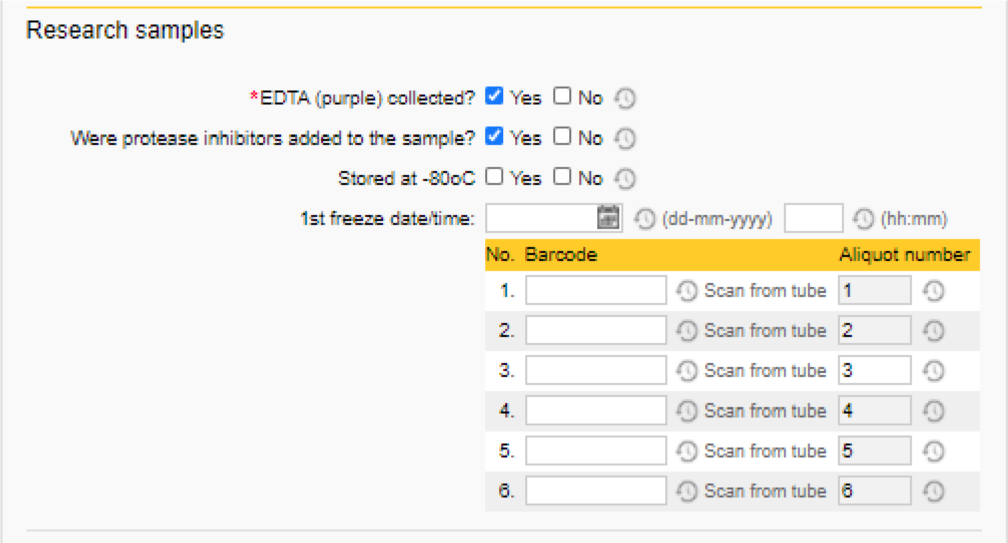

7. Scan in each barcode of the tubes of that type of sample and record the date and time that these tubes will enter the freezer.

9.4 Freezing blood samples

- Once the samples have been logged place the samples in the next available position in the current 0.7 ml FluidX box in a -80°C freezer immediately.
- If positions have been incorrectly recorded previously, ensure these are corrected on the Excel file and on the Sample log.
- The temperature of this freezer should be monitored regularly, in accordance with existing regulations (e.g. HTA).

10. Missing or illegible labels

If labels are missing or unreadable from tubes, the Chief Investigator must be informed within 24 hours and an investigation into labelling procedures and materials started to ensure trial sites are using robust labelling materials; if this is not the case, the labelling policy must be changed and all labelled samples using the previous method must be updated to comply with the new standards within one month. Any samples which cannot be identified due to missing labels must be destroyed (see section 13, **Sample Disposal**).

11. Sample transfer to CI's laboratory

11.1 Arranging a courier collection

All research samples will be transferred to the KCL laboratory in batches using dry ice. The site must liaise with the KCL laboratory before arranging shipment of any samples to ensure someone is available to receive the shipment. Shipment of samples should take place before Wednesday of any given week to ensure samples remain frozen and can be received without compromise in case of delay.

Each site will arrange shipment with courier CitySprint. Dry ice and thermal units are supplied by CitySprint. A **minimum of 24 hours'** notice is required to supply these.

Samples should be sent upon request by the coordinating centre. At the end of the study, all samples should be shipped in a final batch.

Note: ST samples can be delivered by hand to the KCL laboratory. Samples from QCCH and NT will require courier delivery each time.

CitySprint details:

Account name: GSTT – GUARD

Account number: CS201901

Contact number: 020 7880 1115

Contact email: healthct@citysprint.co.uk

Hours of operation: 08:00 – 19:00

11.2 Sample packaging

Packaging material, dry ice and instructions will be supplied by CitySprint for all sites.

NOTE: ST must also pack samples on dry ice for hand delivery.

11.3 Documentation

11.3.1 Shipment labels

Shipment labels will be provided by the courier. If samples are walked from ST to Guy's campus, the outer box must be labelled with the recipient address and person responsible and a contact number.

11.3.2 Shipment logs

The courier will provide a receipt for each shipment. This will be based on the information sent by sites when requesting shipment.

A paper log of all samples shipped must be sent with the samples, and an electronic copy emailed to the receiving site. Please see below for how to ship the sample logs.

11.3.3 Sample logs for shipping

When shipping samples, it is important for a log of all the samples being shipped to be accounted for. Samples will be shipped as boxes, so the **GUARD –Box Shipment Log** needs to be completed with a log of all the box barcodes that have been shipped. If a sample log has a page with samples whose box is not being shipped, please cross these off on the photocopy to indicate that they are not being sent in this shipment. After you have completed this, you will need to scan all of the photocopied logs which are in the boxes that you will ship (check the box barcodes match). The photocopied samples logs will need to be emailed to the receiving site, and a copy printed and added into the shipment box.

11.3.4 Receiving samples

When samples have been received, the contents must be checked as soon as possible against the Shipment Box Form and Samples Logs sent with the shipment to ensure all samples recorded as sent are present. If samples are not present, this must be urgently queried with the shipping site to determine where the discrepancy occurs and whether samples are missing. If the samples cannot be found, this must be escalated, and the Chief Investigator must be informed within 24 hours and an investigation started. In case of damage the courier will be notified.

The Shipment Box Form alongside the photocopies of the Sample Logs must be stored in the GUARD Trial file at the receiving site, documenting the date of delivery, receipt and date and time samples are placed in storage at KCL will be kept. Once the samples are received by the allocated personnel of the receiving site, confirmation will be sent to the shipping site via email.

12. Freezer monitoring

All freezers used to store samples for the GUARD trial must be associated with a temperature log to ensure the integrity of the sample. Please make sure that this system is in place before you begin taking samples for GUARD. These records must be maintained whilst samples are on-site, and a full report sent to the CI once the final batch of samples has been shipped. Please file copies of these records in the ISF. All freezers used for GUARD should be calibrated once per year.

All sites should have a back-up freezer which has room enough to accommodate all samples should there be a freezer failure.

12.1 Freezer information required to be recorded

The following information should be recorded for each freezer that samples for GUARD are kept in:

- Freezer ID
- Location
- Temperature range
- Date of check
- Time of recording
- Initials of recorder (if using a manual system)
- Current temperature
- Minimum temperature
- Maximum temperature
- Any comments

The above is the minimum requirement if no automatic temperature monitor is in place. An automatic temperature monitor is highly favourable; however, if this is not possible, then the freezer must be checked daily to ensure the integrity of the samples.

The temperature excursion log can be found in **GUARD Temperature Excursion Log**.

12.2 Reporting a temperature excursion

If a temperature excursion occurs outside of the acceptable range (all samples for GUARD are required to be kept at -80°C, with an acceptable range of -60°C to -90°C), this could compromise the integrity of the samples. Any temperature excursions must be reported to the trial manager for your site, the Clinical Research Associate, and the Postdoctoral Research Associate (contact details on page 2). You should email them as soon as possible after a temperature excursion, detailing the following information:

- Freezer ID
- Lowest temperature reached
- Duration the freezer was outside of the acceptable range
- Box barcodes of boxes affected
- Were the samples moved to a back-up freezer? If so, how long were these samples outside of acceptable temperature range before they were moved?

The Clinical Research Associate and the Postdoctoral Research Associate will then perform a risk assessment to determine whether sample integrity has been compromised and will record this documentation.

13. Sample Disposal

Samples must be disposed of when a patient withdraws consent and requests sample destruction, or if required so under any other circumstances. This must be done as soon as feasibly possible by a designated person at the site where the samples are stored. **GUARD Sample Disposal Form** must be completed upon disposal of the samples and sent to the coordinating centre for filing.

Samples requiring disposal should be securely placed inside a sharps bin which will be sent for incineration by the site.

14. End of study

14.1 Shipping final samples

Remaining samples at each site will be shipped as a final batch as per section **11**.

14.2 Shipping temperature logs once all samples have been sent

Shipping all the freezer temperature measurements is unnecessary, however, GUARD requires that any temperature excursions must be recorded using the **GUARD Temperature Excursion Log**.

All temperature excursions should be printed and shipped with the final batch as evidence of sample integrity. Please retain copies for your ISF.

Stool Collection Instructions

You have been asked to give a stool sample as part of the GUARD study. This is a very important part of the study, as the different types of bacteria in your gut may help us understand how your body responds to the medication that you are taking for your diabetes during pregnancy.

Donating a stool sample can be done either in the clinic for your Follow Up 2 visit, or at home around the time of your Follow Up 2 visit. You should also be given a food diary, which should be completed around the time of your stool sample, preferably for the 4 days before you give your sample, although we understand that this might not always be possible.

Your stool collection kit should contain the following items:

1. 1 pair of disposable gloves
2. Hystool flushable collection bag with tape pre-attached
3. Sterile specimen pot with attached scoop (your research midwife will have written your GUARD study ID number on this; please do not fill in any details on this label apart from the Date and Time when you collect your sample)
4. Sealed pouch (your research midwife will have attached a label with your GUARD study ID number on this)
5. Plastic bag for waste collection
6. Polystyrene box inside a cardboard box (not pictured)
7. Ice blocks (not pictured) – *please put these in the freezer when you get home to ensure they are cold enough when the courier comes to collect your sample!*

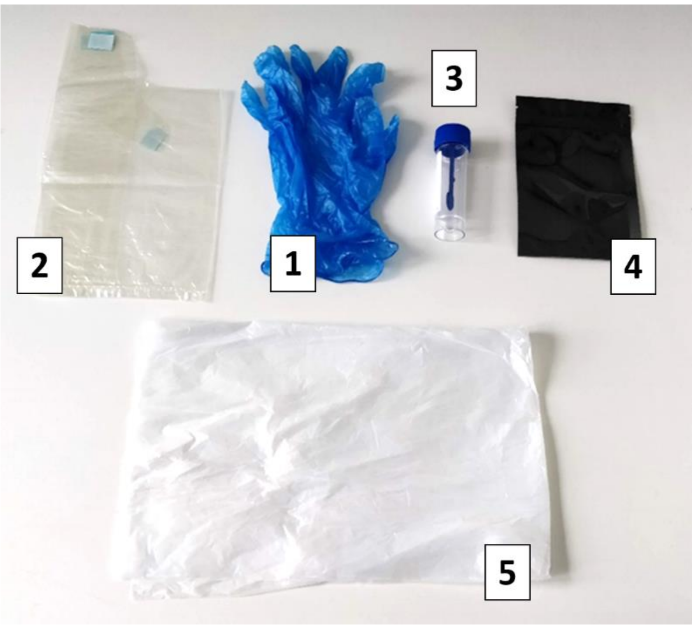

Your label should have been filled in by the midwife when you were given the stool collection kit and should contain your GUARD study ID number. There is space for you to write the date and time on the stool collection pot when you collect your sample. Please make sure you remember to fill this in.

How to collect your stool sample

1. If you need to urinate, please do so before you follow steps 1 to 13. YOU MUST ENSURE THAT YOU DO NOT URINATE AT THE SAME TIME AS COLLECTING YOUR STOOL SAMPLE - this would contaminate the sample and may disintegrate the Hystool bag used for collection.

2. Put on the disposable gloves (item 1)

3. Open up the Hystool stool collection bag (item 2) and firmly press the tape onto the toilet seat so that the bag sits towards the rear of the toilet bowl.

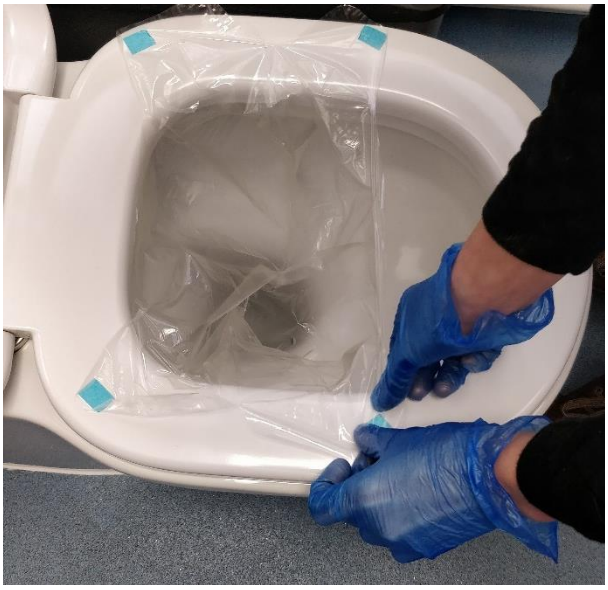

4. Place a folded piece of toilet roll in the bottom of the Hystool bag.

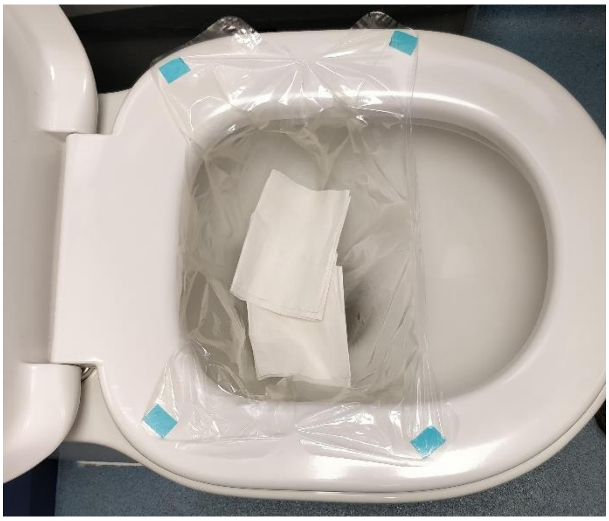

5. Pass your stool. The stool should land inside the Hystool bag. REMEMBER, YOU MUST ENSURE THAT YOU DO NOT URINATE AT THE SAME TIME.

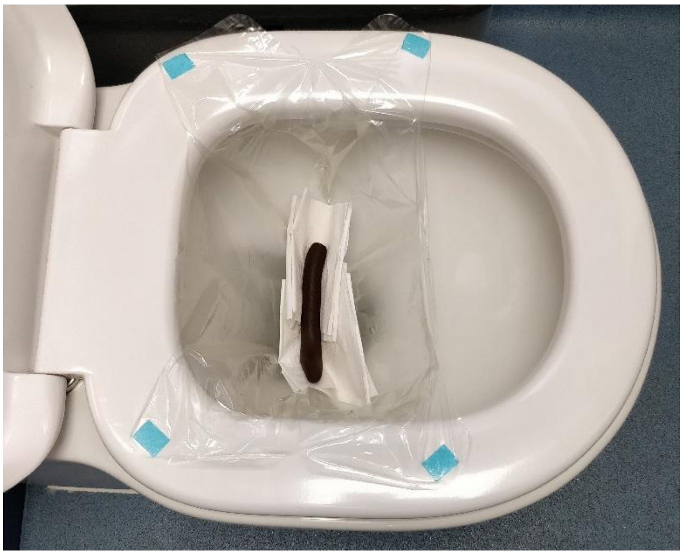

6. Still wearing your gloves, open the sterile specimen pot (item 3) and, being careful not to touch anything but the stool sample, scoop up the stool sample into the pot. You can place your hand underneath the Hystool bag to help stabilise the bag whilst you scoop up your sample. Aim to fill around 1/4 of the pot with your stool sample.

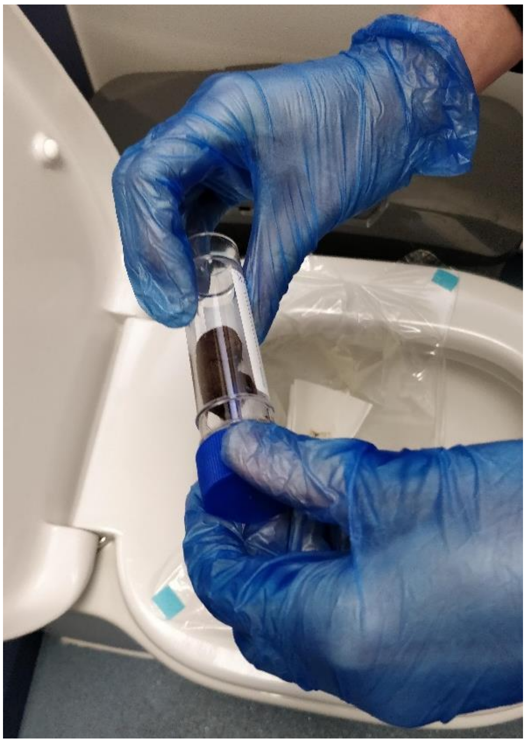

7. Screw the lid on tightly on the pot.

8. Fill in the label on the collection pot with the time and date of your sample collection. Your GUARD study ID number should already have been filled in by the midwife. If this is not the case, please contact the midwife so that she can tell you the study number for you to write on the label. Please fill in the <u>date</u> and <u>time</u> that you have collected your sample, but no other information.

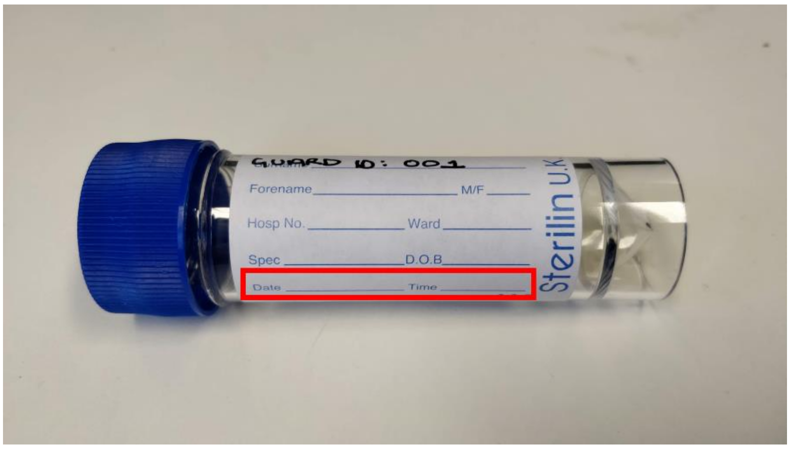

9. Place the stool collection pot in the sealable pouch (item 4), making sure the seal is tightly closed.

10. Pull up the tape on the Hystool collection bag and lower it into the toilet. The bag should dissolve with a few minutes, and you can flush the toilet as normal.

11. Take off your disposable gloves and place them in the black bag (item 5) provided. This can be placed in your normal waste.

12. <u>Immediately</u> place the pouch containing the stool sample into your freezer. If you haven't already placed the ice blocks in the freezer, please do so now to make sure they keep your sample cool when it is shipped.

13. Contact your midwife to tell them that you have collected your stool sample and let them know when would be a good time for the sample to be collected. They will arrange a courier (CitySprint) to come and collect your stool sample at a time that is convenient to you. Until then, please leave the sample in your freezer.

14. The CitySprint courier will arrive at the pre-arranged time. Please place your sample into the polystyrene box and make sure it is covered by the ice blocks as much as possible. Place the polystyrene lid on the box, fold the flaps of the cardboard box over the polystyrene box to secure the sample. The courier will then take this to the laboratory at your hospital.

Thank you for your time!

GUARD Laboratory Manual Training Log

SITE NAME:

I confirm that I have read and agree to follow the GUARD Laboratory Manual, version 3.0, dated 18-06-2021.

Name	Role	Signature	Date

GUARD - Maternal Blood Sample Collection Form

Please first confirm eligibility and unique participant database ID.

ID: ___________ **Initials:** ___________ **Date of Visit:** ___________

Please collect the following bloods from each patient in the recommended draw order depending upon the study visit:

Trial Bloods (For Clinical Lab):

	Taken?	Time taken
**Baseline**	☐ 1 x 3.5 ml Serum (SST), gold top vacutainer	___________
	☐ 1 x 3.5 ml Serum (SST), gold top vacutainer	___________
	☐ 1 x 6 ml EDTA, lavender top vacutainer (onto ice) – *only take if no HbA1c result available within the past 3 weeks*	___________
**Follow-Up 1**	None required	
**Follow-Up 2**	☐ 1 x 4 ml FO, grey top vacutainer	___________
	☐ 1 x 3.5 ml Serum (SST), gold top vacutainer	___________
	☐ 1 x 3.5 ml Serum (SST), gold top vacutainer	
	☐ 1 x 6 ml EDTA, lavender top vacutainer (onto ice) - *only take if no HbA1c result available within the past 3 weeks*	___________

Research Bloods (For Research Lab):

Please notify the research technician who will be processing the bloods, so that they can begin centrifugation 30 minutes after the bloods are taken and send this form with the bloods. For GUARD-MEC research samples, please ensure that the GUARD-MEC Blood Sample log is filled in instead.

	Taken?	Time taken
**Baseline**	☐ 1 x 6 ml EDTA, lavender top vacutainer (onto ice) – **add protease inhibitors**	___________
**Follow-Up 1**	☐ 1 x 6 ml EDTA, lavender top vacutainer (onto ice) - **add protease inhibitors**	___________
**Follow-Up 2**	☐ 1 x 5 ml Serum (SST), gold top vacutainer	___________
	☐ 1 x 6 ml EDTA, lavender top vacutainer (onto ice) - **add protease inhibitors**	___________

Please complete the sample pages for this visit on the database after the samples have been sent to the lab. Please file this form in the GUARD Lab Folder after processing

GUARD – Meconium Collection Form

.

ID: __________ **Initials:** __________

Clinical Midwives:

Please put the baby's first meconium nappy in the bag provided and fill in the date and time of collection below. Place in the research fridge if possible, and if Monday-Friday 9-5 please call the research midwives to collect. Research midwives ext 83570 or 83634

Nappy collection:

Date: ___/___/______ Time: _______:_______

Research midwives:

Is the nappy sample more than 4 hours old? If so, please do not collect a meconium sample as the integrity of the sample has likely been compromised.

If the nappy sample is less than 4 hours old, please scrape the meconium from the nappy and place in a 5mL FluidX tube and bring immediately to the sample processing team.

Meconium scraped into vial:

Date: ___/___/______ Time: _______:_______

Please complete the sample pages for meconium collection after the samples have been sent to the lab.

Please file this form in the GUARD Lab Folder after processing.

GUARD - Maternal Blood Sample Log

GUARD ID	Initials	Visit*	Date and Times				Number of Aliquots		Location†							Logged in MedSciNet?	Processed by (initials)	Comments
			Date Taken	Time Taken	Time Spun	Time Frozen	Serum	EDTA Plasma	Box Barcode	First Position (Row/Col)	Last Position (Row/Col)	Freezer	Shelf	Rack	Position			

*Visits: B = Baseline; F1 = 1st Follow up (32w); F2 = 2nd Follow up (36w); M0 = MEC Fasted; M15 = MEC 15 minutes; M1H = MEC 1 hour; M2H = MEC 2hour

†Each box contains A-H rows and 12 columns. Freezer racks have 4 trays and hold 6 boxes per tray, 24 boxes per rack.

GUARD - Cord Blood Sample Log

GUARD ID	Initials	Date and Times				Number of aliquots			Location†							Logged in MedSciNet?	Processed by (initials)	Comments
		Date Taken	Time Taken	Time Spun	Time Frozen	Arterial Serum	Venous Serum	Mixed Serum	Box Barcode	First Position (Row/Col)	Last Position (Row/Col)	Freezer	Shelf	Rack	Position			

†Each box contains A-H rows and 12 columns. Freezer racks have 4 trays and hold 6 boxes per tray, 24 boxes per rack.

GUARD - Blood Spot Sample Log

GUARD ID	Initials	Type of Sample (Cord Blood / Neonatal Heel Prick)	Date and Times				Number of spots*			Location†					Logged in MedSciNet?	Processed by (initials)	Comments
			Date Taken	Time Taken	Date Frozen	Time Frozen	Arterial Blood	Venous Blood	Mixed Blood	Box Barcode	Freezer	Shelf	Rack	Position			

†Each storage box has rows A-D and 4 columns. *If a cord blood sample was used for the blood spot, please indicate whether the number and sample types. If a neonatal heel prick was taken for the blood spot, please fill in "Mixed Serum" column only.

GUARD – Maternal Stool Sample Log

GUARD ID	Initials	Date and Times						Aliquot Barcode	Storage Location†						Logged in MedSciNet?	Processed by (initials)	Comments
		Date Produced	Time Produced	Setting (Home or Clinic)	Confirmed frozen at home?	Date Received in Lab	Time Frozen in Lab at -80		Box Barcode	Position (Row/Col)	Freezer	Shelf	Rack	Position			

†Each storage box has rows A-D and 4 columns

GUARD – Meconium Sample Log

GUARD ID	Initials	Date and Times				Aliquot Barcode	Location†						Logged in MedSciNet?	Processed by (initials)	Comments
		Date Taken	Time Taken	Time Processed	Time Frozen		Box Barcode	Position (Row/Col)	Freezer	Shelf	Rack	Position			

GUARD –Box Shipment Log

Shipping site: ___________________________ Date of shipment: ____ /____ /________ Time samples removed: ____ : ____ Staff initials: _____

Receiving site: ___________________________ Date of receipt: ____ /____ /________ Time of receipt: ____ : ____ Staff initials: _____

Received at correct temperature? ______

Shipping site					Receiving site			
**Box barcode**	**Type of samples ***	**Photocopy of relevant sample log pages for box barcode taken?** (Tick when completed)	**Original storage location ✝**	**Comments**	**Box contained in shipment?**	**Samples present in shipment?**	**New storage location ✝**	**Comments**

* (b = maternal blood, f = faeces, m = meconium, cb = cord blood); ✝ (freezer = F, shelf = S, rack = R, position = P) e.g. "F1, S2, R3, P4"

GUARD Temperature Excursion Log

Site name	Fridge/Freezer ID	Date	Time	Duration of temperature excursion	Highest temperature reached (°C)	Samples affected (please detail box barcode)	Samples moved to a back-up fridge/freezer?	Time / temperature when samples were moved	New sample location	Date samples were returned	Did sample assessment produce results within the normal ranges?

* (b = maternal blood, f = faeces, m = meconium, cb = cord blood); ŧ (freezer = F, shelf = S, rack = R, position = P) e.g. “F1, S2, R3, P4”

GUARD Sample Disposal Form

To be completed by the person undertaking the disposal of relevant material for research. This document should be stored by the Chief Investigator and a copy added the HTA departmental file for reference.

**Study Title**	Randomised controlled trial of **G**estational treatment with **U**rsodeoxycholic **A**cid compared to Metformin to **R**educe effects of **D**iabetes mellitus
**REC number**	
**Chief Investigator or custodian of collection**	
**Sample barcode(s)**	
**Type and amount of sample to be disposed**	
**Date of sample collection**	
**Date and time of disposal**	
**Reason for disposal**	
**Method of disposal.** Was this in accordance with any conditions of the patient's consent (if applicable)	
**Person responsible for disposal (name and signature)**	Name: Signature:
**Additional details/comment**	
**Disposal authorised/witnessed by?**	Name: Signature:

* (b = maternal blood, f = faeces, m = meconium, cb = cord blood); † (freezer = F, shelf = S, rack = R, position = P) e.g. "F1, S2, R3, P4"
