# Supplementary material for: A multi-centered trial investigating gestational treatment with ursodeoxycholic acid compared to metformin to reduce effects of diabetes mellitus (GUARD): a randomized controlled trial protocol
Source: Trials. 2022 Jul 19;23:571. doi: 10.1186/s13063-022-06462-y (PMC9295112; doi:10.1186/s13063-022-06462-y)
Supplement: Supplementary file 2 — Additional file 2. [file 13063_2022_6462_MOESM2_ESM.pdf]

KING'S College LONDON

NHS Guy's and St Thomas' NHS Foundation Trust

GUARD Patient Information Sheet

Randomised controlled trial of **G**estational treatment with **U**rsodeoxycholic **A**cid compared to Metformin to **R**educe effects of **D**iabetes mellitus

We would like to invite you to take part in our study

- We would like to invite you to take part in a research study. We appreciate this is a difficult time to think about research. Before you decide it is important that you understand why the study is being done and what it will involve.
- Please discuss the study with family and friends.
- One of our team will go through the information with you at your next appointment, and answer any questions you may have.

PART 1 – WHAT IS INVOLVED

What is the purpose of the study?

Gestational diabetes mellitus (GDM) is a condition that develops in pregnancy and is caused by your blood sugar (glucose) level being high. It happens because your body cannot produce enough insulin (a hormone that helps control blood sugar level) to meet the extra needs in pregnancy. This condition often gets better after giving birth.

GDM needs treating. Most women with GDM have normal pregnancies with healthy babies. But sometimes it can cause problems for mother and/or baby during pregnancy or birth. This could range from developing high blood pressure, having your baby early, your baby growing larger than usual, or your baby developing low blood glucose after birth, amongst others. Having good control of blood glucose in pregnancy can reduce the chances of having problems. Many women can control their blood glucose levels through what they eat and by increasing physical activity. If these do not work, you may need to take tablets. Metformin is the most commonly used medication to treat GDM when dietary changes have not helped. If this does not help either, then you may need to take insulin.

Metformin is an effective treatment in people with Type 2 diabetes. However, it does not always effectively control glucose levels in pregnancy. We know that metformin crosses the placenta. There is not a lot of information into the long-term effects on metformin on the child.

UDCA is a drug that is used to treat women with another liver problem in pregnancy called intrahepatic cholestasis of pregnancy (ICP). Although this drug is not licensed for use in pregnancy (as is the case for metformin), it has been widely used in ICP for many years. There is evidence from other research studies to suggest that UCDA may be good at controlling glucose in both the mother and the baby.

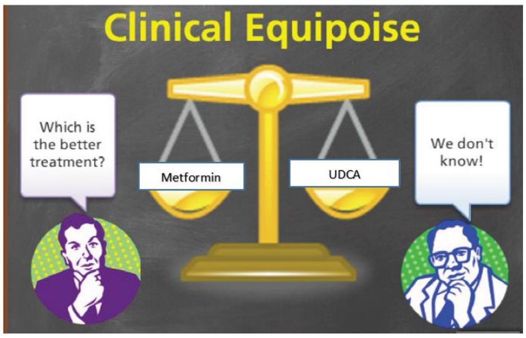

As we don’t know which one is better, in this study we will compare the effects of both treatments (metformin vs UDCA) to control glucose levels, pregnancy and birth outcomes. The study will also be asking women about how easy and acceptable it is to take the medications, and about their quality of life. We are inviting 158 women to participate.

This study is a pilot. This means that if the information we get is positive, we plan to do a much larger study later on.

Why have I been invited, and do I have to take part?

You have been invited to take part because you have been diagnosed with GDM and you might need to take tablets. If you do not need tablets, you will not be able to take part. Whether you decide to take part or not is entirely up to you. Your decision will not affect the care you or your baby receive in any way. If you agree to take part, you are free to withdraw at a later stage without giving a reason.

It is important that you understand what is happening at every stage. At each visit you will be given as much time as you need to discuss any concerns.

What are the drugs being tested?

We are comparing two medicines:

- **Metformin**: a drug used to treat type 2 diabetes. It works by reducing the amount of glucose your liver releases into your bloodstream. It also helps your body respond better to insulin (the hormone that controls the level of glucose in blood).

- **Ursodeoxycholic acid**: this tablet is a form of a naturally occurring bile acid that is present in your body in very small amounts. It has been shown to reduce the blood glucose in people with type 2 diabetes who also have fatty liver disease. Small studies have suggested it also improves blood glucose in pregnancy.

You will take only one of these two drugs. Both drugs are taken in the morning and the evening usually with meals. If the tablets you are prescribed do not work you may need further treatment, but this will be discussed with you by your doctors and midwives.

What will happen to me if I take part?

Below is a chart that shows what each of the study visits involves:

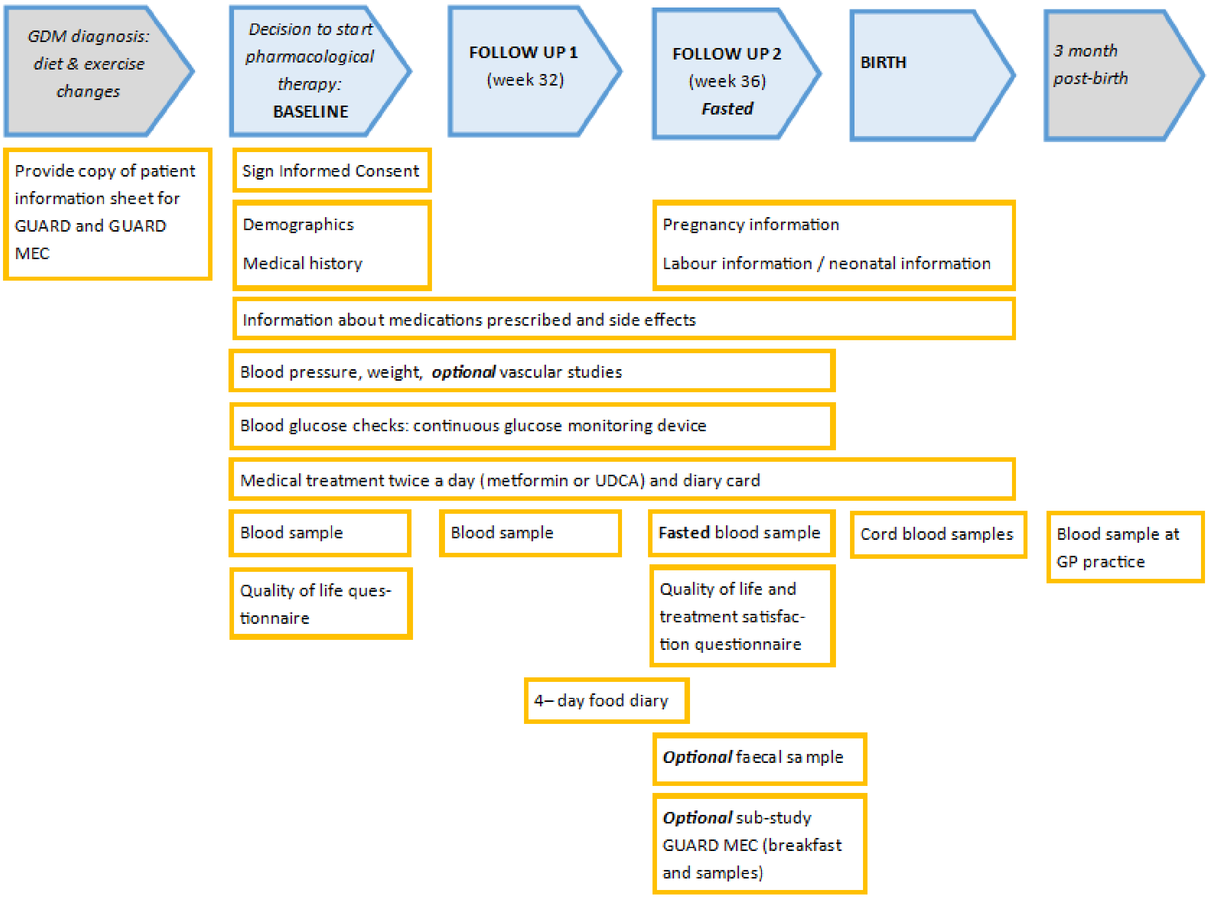

Once you have had time to think about the study, and if you agree to take part, we will ask you to sign a consent form. As this is a randomised study, neither you nor the doctor will decide which treatment will be given. We will enter some details about you in a computer (such as your weight and height), which will then allocate you to a treatment randomly, in the same way of tossing a coin. This study is 'open label', which means that both you and your doctor will know which treatment you have been prescribed.

We will see you at three different visits (Baseline: week 27-31, Follow up 1: week 32, and Follow up 2: week 36 –weeks into your pregnancy-), and we will collect information about your labour. All of these visits will happen alongside your clinical care visits, where possible. If you are not due to come into the hospital for your appointment, then the research midwife may offer to see you at home instead. Prior to the visit the research midwife will call you to confirm whether you or anyone in your household has COVID-19 symptoms or are self-isolating. During the home visit, adequate measures will be in place, such as use of Personal Protective Equipment by yourself and the research midwife, following hospital guidelines, to minimise the risk of exposure to Covid-19. Three months after you have had your baby, we will contact your GP to obtain a copy of the results of a blood test that you

will have had as part of your routine clinical care. This is undertaken for all women who have had GDM.

- At your first GUARD study visit, we will ask you questions and details about your medical and family history and pregnancy health. We will collect information about previous blood results from NHS computer systems. This may be done over the telephone if you are not coming into the hospital.

- We will ask you to complete 2 different questionnaires at two different visits. One is about your quality of life. The other is about how happy you are with the treatment. Each questionnaire will take about 5 minutes to complete. We will also ask you to keep note of everything you eat and drink for 4 days around the time of the Follow up 2 visit. We will give you a diary card with instructions on how to do it.

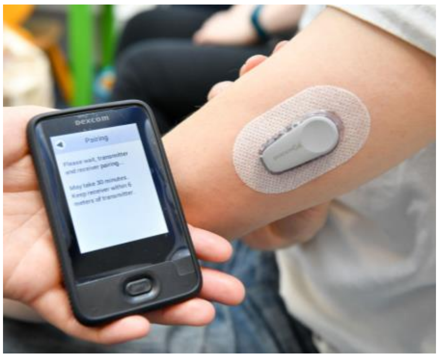

- We will ask you to wear a continuous glucose monitor (CGM). Your clinical care team or the researchers will insert this. The device has two parts, a sensor and a transmitter. The sensor is applied using an applicator to insert the sensor wire painlessly under your skin, usually at the top of your arm. This is held in place using a dressing similar to a plaster. The transmitter is then clicked into the plastic holder that is on the dressing. It is painless and once in place you will not feel it. This sensor measures your glucose levels 24/7. We will ask you to wear this device three times for 10 days at each time, and to remove it after this time. You can take it off yourself by removing the adhesive patch (which you need to return to us). We will download these data every time you return into the clinic. We will ask you to return this device to us when you come into hospital to have your baby. You won't be able to see your blood glucose levels measured by this method (this is called "blinded"), but we can give you a print out or email it you at the end if you want. This device is not used in NHS practice routinely, where blood glucose is measured with finger pricks. You will also have to do the finger pricks as per standard of care (this is not part of the research).

- We would like to take blood samples at all three visits, in addition to those needed as part of your routine care. We will ask you not to eat or drink for at least 3 hours before your 'week 36' visit (during this time you can drink water/black coffee/black tea). You do not need to fast for your other two study visits. .

  After the birth of your baby we would like to collect a blood sample from the umbilical cord or alternatively a baby heel prick if the cord blood collection is missed. This will not affect delayed cord clamping or any other tests that are required (for example if you are rhesus negative). We will also collect some information about your labour and birth and your baby's health in hospital.

- You will be prescribed enough tablets to last until the following visit. Each time you come we would like you to bring any remaining tablets.

  As part of the GUARD study, you will be asked if you will consider taking part in two extra parts which are **optional**: the vascular study and stool samples

- Vascular study: After measuring your height we would like to measure your blood pressure using a special device called the arteriograph. The Arteriograph is primarily the same as a usual 24 hour BP measuring device but also estimates how well the main central arteries of the body (the aorta and

branches) are functioning. It will be put on at the start of your visit & taken off at the end, to allow multiple readings a few minutes apart. The cuff on your arm also measures how long the first pumping wave of blood from the heart takes to be reflected back from the more peripheral blood vessels. That time indicates how stiff those central blood vessels are. The cuff is inflated just a bit more than usual so it can feel tighter and takes a little longer to deflate as it also measures over 6 to 12 heart beat cycles, depending how fast the heart is beating (your ‘heart or pulse rate’).

- Stool sample: We may ask you to give us a stool (poo) sample when you are about 36 weeks pregnant. This is completely optional and you don’t have to do it unless you are happy to do so. You could do this at the clinic or at home, where you would have to freeze the pot immediately. This sample would be put in a special container and collected from your home by a courier.

We may also ask you if you want to participate in a sub-study of GUARD called GUARD MEC. This involves having a breakfast in hospital and donating blood samples. Not all participating hospitals will carry out the GUARD MEC sub-study. Your local research team can advise if this is available at your hospital and give you a separate information leaflet.

There is a rare possibility that your analysis produce an unexpected result (such as high levels of lipids following your fasting blood sample). This may need further investigation or monitoring. If this happens, we will discuss this with you. If necessary, we will provide any support that you may need, such as advising you to contact your GP, or arranging follow-up tests and/or treatment.

What are the possible risks and benefits of taking part?

Participating in this study may not have any direct benefits to you. But we hope that this study may provide information that helps us improve care for women with GDM and their babies in the future.

Both drugs are prescribed for pregnant women regularly in clinical practice and doctors believe they are both safe. However extra treatment may be necessary if your blood glucose levels remain high.

These drugs do have some possible side effects that some women may have:

- Metformin: one in ten people will report feeling sick, vomiting, stomach cramps, diarrhoea and loss of appetite. One in about fifty people will report changes in taste. These side effects usually pass very quickly. One in about ten thousand people will have skin reactions, lactic acidosis and liver effects. These rare side effects get better within a few days to weeks of stopping the drug.
- UDCA: One in about fifty people will have diarrhoea. Others have reported feeling sick, vomiting, stomach cramps, but we don’t know how often. One in about ten thousand people will have a skin rash. These side effects all get better within a few days to weeks of stopping the drug.

While on study you will need to avoid the following medicines:

- If you are in the **Metformin** group: *trimethoprim and vancomycin.*
- If you are in the **UDCA** group: *charcoal, colestyramine, colestipol or antacids containing aluminium hydroxide and/or smectite (aluminium oxide).*

In regards the Glucose Continuous Monitoring device, the most common risk is a minor skin reaction to the adhesives used to keep the CGM in place. Minor itch, redness, bleeding, and bruising at CGM insertion sites may occur.

If you experience any side effects or have any problems please contact the research team.

Taking part in the study may mean that you spend extra time at the hospital, but whenever possible you will be seen at the same time as your routine clinical appointments. If you are not due to attend the hospital, the research midwife may be able to see you at home as described above.

What will happen after my participation in the study finishes?

Once you have given birth you will stop taking your medication. When you are discharged from hospital your participation in the study will end (although we will contact your GP 3 months later to obtain the result of the blood sample).

However we might want to find out about your health and your child's health in the future. This is to understand better the effects of the drugs in the long-term, as both drugs can change the microbes in the gut and this may result in changes in metabolism. We will ask you for your permission to contact you again in few years' time, when you will be asked if you are willing to participate in a future study.

PART 2 – FURTHER INFORMATION

What will happen to my samples?

At each study visit we will ask for a blood sample (up to a maximum of 28 ml of blood, which is roughly 2 tablespoons), some of this will be for routine antenatal tests and others for research. Following delivery of your placenta we would like to take a small blood sample from the umbilical cord (approx. 4 ml, or one teaspoon). We will also take a bit of the first poo of your baby, called meconium.

The laboratory analyses will be done at King's College London and possibly at other hospitals and universities within and outside the EU. It is possible that academic and commercial collaborations will be developed to analyse samples. Your samples will be stored without your personal details. Only the doctors at your hospital will be able to link these samples to you.

Leftover samples may be made available for future research studies as long as you agree to this. We don't have a lot of information about what these samples will be used for, but we will prioritize research into Women and Children's Health, with investigators who have received the necessary approvals. This research could be in or outside the UK, and could be with academic or commercial partners. Nobody will know who you are from your samples, so you will not receive any other information or results.

What if relevant new information becomes available?

Should new information become available the researchers will discuss this with you. You can then decide if you want to continue in the study. It may be that the doctors and midwives decide it would be better for you to stop being part of the study. If this happens, they will discuss this with you.

What if I decide I do not want to carry on with the study?

Whether you decide to take part in the study or not is entirely up to you. Your decision will not affect the care you or your baby receive in any way. If you agree to take part, you are free to withdraw at a later stage, without giving a reason, although you may be asked if you mind us collecting details about your labour from your NHS records. Again, it is entirely up to you if you agree to this.

How will we use information about you?

We will need to use information from you for this research project. This information will include your name, date of birth, contact details, postcode, hospital number and possibly email address. People will use this information to do the research or to check your records to make sure that the research is being done properly.

Dexcom is the company who provides the glucose monitors. They will receive information about your blood glucose, but no other personal information.

People who do not need to know who you are will not be able to see your name or contact details. Your data will have a code number instead. We will keep all information about you safe and secure.

Once we have finished the study, we will keep some of the data so we can check the results. We will write our reports in a way that no-one can work out that you took part in the study.

What are your choices about how your information is used?

- You can stop being part of the study at any time, without giving a reason, but we will keep information about you that we already have. If you wish for us to destroy the biological samples that we have not analysed yet, please let us know.
- If you choose to stop taking part in the study, we would like to continue collecting information about your health from your hospital. If you do not want this to happen, tell us and we will stop.
- We need to manage your records in specific ways for the research to be reliable. This means that we won't be able to let you see or change the data we hold about you.
- If you agree to take part in this study, you will have the option to take part in future research using your data saved from this study.

Where can you find out more about how your information is used?

You can find out more about how we use your information:

- at www.hra.nhs.uk/information-about-patients/
- https://www.guysandstthomas.nhs.uk/research/patients/use-of-data.aspx.
- www.kcl.ac.uk/research/support/research-ethics/kings-college-london-statement-on-use-of-personal-data-in-research
- by asking one of the research team
- by sending an email to dpo@gstt.nhs.uk

What if there is a problem?

If you have a concern about any aspect of this study, you should ask to speak to the doctor or midwife who leads the study in your hospital, and they will do their best to answer your questions. If you remain unhappy you can make a formal complaint through the NHS complaints procedure. Details can be obtained through your hospital's Patient Advisory Liaison Service (PALS).

All staff involved in the study hold professional indemnity to work within their Trust. The Sponsors will at all times maintain adequate insurance in relation to the trial through their own professional indemnity (Clinical Trials) and no fault compensation. In the event that something does go wrong and you are harmed during the research you may have grounds for legal action for compensation against Guy's and St Thomas' NHS Foundation Trust London and/or King's College London, but you may have to pay your legal costs.

The normal National Health Service complaints mechanisms will still be available to you (if appropriate).

Who is organising and funding the research?

The research has been funded by the J. P. Moulton Charitable Foundation and it is being sponsored by King's College London and Guy's and St Thomas NHS Foundation Trust. The Chief Investigator is Professor Catherine Williamson, from the Division of Women and Children's Health, KCL.

Who has reviewed the study?

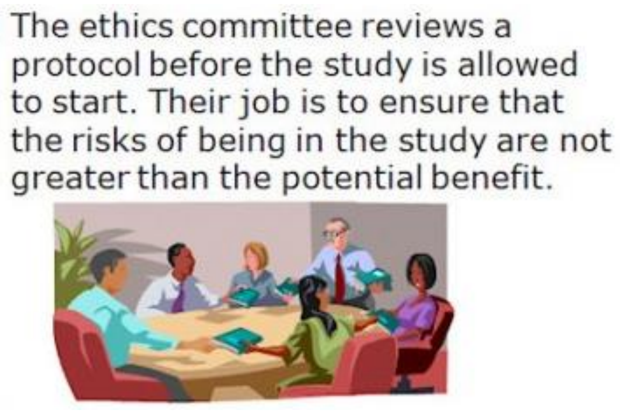

Before any research study can go ahead, it must be checked by an independent Research Ethics Committee, the Health Research Authority (HRA) and the Medicines and Healthcare products Regulatory Agency (MHRA) to make sure that the research is fair and transparent and that your rights and wellbeing will be protected. The London Westminster Ethics Committee has reviewed and approved this study in the UK. We will send reports to those institutions once a year to let them know how the research is going. The Research & Development team at your hospital must also approve the study.

What will happen to the results of the research?

Information about this research and its results will be published in a research registry, Clinicaltrials.gov. All clinical trials need to be registered in public databases to make the research transparent. The study results will be presented at meetings and published in medical journals but you will not be identified. After the study has ended, we will send a newsletter with the study results to your research team. If you wish, we can send you an email with the study results. We will ask you to provide your email address. Results will also be disseminated through relevant patient groups (e.g. Tommy's).

Details of local team

Name of Principal Investigator: Professor Catherine Williamson

Contact information for Principal Investigator: catherine.williamson@kcl.ac.uk

Name of local midwife: Holly Lovell

Contact information for local midwife: holly.lovell@gstt.nhs.uk 0207 188 3570/ 3634

Contact details for local PALS: pals@gstt.nhs.uk

Chief Investigator: Professor Catherine Williamson catherine.williamson@kcl.ac.uk

Guy's and St Thomas' NHS Foundation Trust

PATIENT CONSENT FORM

PART 1 of 2: Use this page ONLY if consenting remotely: Initial Patient Consent

GUARD: Randomised controlled trial of **G**estational treatment with **U**rsodeoxycholic **A**cid compared to Metformin to **R**educe effects of **D**iabetes mellitus

Patient number: ___ ___ ___ ___

Please initial box

1. I confirm that I have received a personal copy of, and have read and understood the Patient Information Sheet Version 2.1 dated 16/03/21 for the above study and have had the opportunity to ask questions and discuss the study. I have been given a copy of the Patient Information Sheet to keep. ☐

2. I understand that my participation in this study is voluntary and that I am free to withdraw at any time, without giving any reason, without my medical care or legal rights being affected. ☐

3. I understand that sections of any of my medical notes and data collected during the study may be looked at by responsible individuals involved in this study, or by regulatory authorities, King's College London, Guy's and St Thomas' NHS Foundation Trust, and the research and development department where it is relevant to my taking part in this research. I give permission for these individuals to have access to my records. I understand that my personal data will be processed and stored securely in compliance with the Data Protection Act 2018 and the General Data Protection Regulation. ☐

4. I agree to my General Practitioner being informed of my participation in the study and to share relevant data with the study team. ☐

5. I agree to the collection, processing, storage and analysis of blood samples, including umbilical cord samples (or baby foot prick) and meconium from my baby after birth. ☐

6. ***Optional:*** I agree to relevant personal details being retained by the local research team and used so that I can be contacted over the next few years for a further study into the follow-up of my baby. ☐ YES ☐ NO ☐

7. ***Optional:*** I would like to receive a copy of the study results by email. ☐ YES ☐ NO ☐

8. ***Optional:*** I agree to provide faecal samples at week 36 and for my address to be shared with the courier for collection of the sample. ☐ YES ☐ NO ☐

9. ***Optional:*** I agree to participate in the 'Vascular studies' for the measurement of blood pressure. ☐ YES ☐ NO ☐

10. ***Optional:*** I agree to my samples being stored for use in future approved research studies, which could be in or outside of the UK, and could be of commercial nature. ☐ YES ☐ NO ☐

11. **I agree to take part in the above study** ☐

Name of Patient	Date	Time	Signature

The investigator will sign Part 2 of this consent form during the telephone call (only for remote consenting).

When completed: 1 copy for patient; 1 copy for medical notes; 1 (original) to be kept in investigator site file

PATIENT CONSENT FORM

Guy's and St Thomas' NHS Foundation Trust

PART 2 of 2: Use this page for face-to-face consenting and for remote consenting. Investigator and Patient Consent

GUARD: Randomised controlled trial of **G**estational treatment with **U**rsodeoxycholic **A**cid compared to Metformin to **R**educe effects of **D**iabetes mellitus

Patient number: ___ ___ ___ ___

Please initial box

1. I confirm that I have received a personal copy of, and have read and understood the Patient Information Sheet Version 2.1 dated 16/MAR/21 for the above study and have had the opportunity to ask questions and discuss the study. I have been given a copy of the Patient Information Sheet to keep. ☐

2. I understand that my participation in this study is voluntary and that I am free to withdraw at any time, without giving any reason, without my medical care or legal rights being affected. ☐

3. I understand that sections of any of my medical notes and data collected during the study may be looked at by responsible individuals involved in this study, or by regulatory authorities, King's College London, Guy's and St Thomas' NHS Foundation Trust, and the research and development department where it is relevant to my taking part in this research. I give permission for these individuals to have access to my records. I understand that my personal data will be processed and stored securely in compliance with the Data Protection Act 2018 and the General Data Protection Regulation. ☐

4. I agree to my General Practitioner being informed of my participation in the study and to share relevant data with the study team. ☐

5. I agree to the collection, processing, storage and analysis of blood samples, including umbilical cord samples (or baby heel prick) and meconium from my baby after birth. ☐

6. ***Optional:*** I agree to relevant personal details being retained by the local research team and used so that I can be contacted over the next few years for a further study into the follow-up of my baby. ☐ YES ☐ NO ☐

7. ***Optional:*** I would like to receive a copy of the study results by email. ☐ YES ☐ NO ☐

8. ***Optional:*** I agree to provide faecal samples at week 36 and for my address to be shared with the courier for collection of the sample. ☐ YES ☐ NO ☐

9. ***Optional:*** I agree to participate in the 'Vascular studies' for the measurement of blood pressure. ☐ YES ☐ NO ☐

10. ***Optional:*** I agree to my samples being stored for use in future approved research studies, which could be in or outside of the UK, and could be of commercial nature. ☐ YES ☐ NO ☐

11. **I agree to take part in the above study** ☐

____________________	__________	________	______________
Name of Patient	Date	Time	Signature
____________________	__________	________	______________
Name of Investigator	Date	Time	Signature

When completed: 1 copy for patient; 1 copy for medical notes; 1 (original) to be kept in investigator site file
